# Efficacy of Internet-Based Acceptance and Commitment Therapy for Depressive Symptoms, Anxiety, Stress, Psychological Distress, and Quality of Life: Systematic Review and Meta-analysis

**DOI:** 10.2196/39727

**Published:** 2022-12-09

**Authors:** Areum Han, Tae Hui Kim

**Affiliations:** 1 Department of Occupational Therapy University of Alabama at Birmingham Birmingham, AL United States; 2 Department of Psychiatry Yonsei Wonju Medical College Wonju Republic of Korea; 3 Department of Psychiatry Wonju Severance Christian Hospital Wonju Republic of Korea

**Keywords:** acceptance and commitment therapy, anxiety, depression, internet-based intervention, meta-analysis, psychological distress, quality of life, stress, systematic review

## Abstract

**Background:**

Acceptance and commitment therapy (ACT) is an empirically supported transdiagnostic approach that involves mindfulness processes and behavior change processes for valued living.

**Objective:**

This systematic review and meta-analysis of randomized controlled trials (RCTs) aimed to assess the efficacy of internet-based ACT (iACT) for depressive symptoms, anxiety, stress, psychological distress, and quality of life (QoL).

**Methods:**

PubMed, CINAHL, PsycINFO, and SCOPUS databases were searched to identify relevant RCTs published up to June 5, 2021. The included RCTs were assessed using the Cochrane Collaboration risk-of-bias tool. The use of either a random effects model or fixed effects model was determined using I2 statistic values for heterogeneity. Subgroup analyses were conducted according to the type of control group, the use of therapist guidance, delivery modes, and the use of targeted participants, when applicable.

**Results:**

A total of 39 RCTs met the inclusion criteria. Meta-analyses found small effects of iACT on depressive symptoms, anxiety, stress, psychological distress, and QoL at the immediate posttest and follow-up. There was no significant effect of iACT on stress at follow-up. Subgroup analyses showed small to medium effects of iACT on all the outcomes at the immediate posttest and follow-up compared with the passive control groups. In contrast, subgroup analyses that compared iACT with active control groups found no differences between groups on stress, psychological distress, and QoL at the immediate posttest or on depressive symptoms, anxiety, and stress at follow-up. In addition, subgroup analyses conducted according to the use of therapist guidance, delivery modes, and the use of targeted participants found no statistically significant subgroup differences among studies in all the outcomes, except for the subgroup difference among studies according to the use of targeted participants for depressive symptoms at the immediate posttest (ie, a statistically significant, larger effect of iACT when studies targeted people with depressive symptoms). The overall risk of bias across the studies was unclear.

**Conclusions:**

The findings of this study contribute to the body of evidence regarding the effects of iACT on depressive symptoms, anxiety, stress, psychological distress, and QoL and may be applicable in any population, as ACT is a transdiagnostic approach. Few studies have compared iACT with active control conditions, especially for stress and psychological distress at the immediate posttest and follow-up. In addition, the active control conditions varied among the included studies. Further high-quality studies are needed to better understand whether iACT is comparable or superior to other evidence-based interventions, such as cognitive behavioral therapy, in decreasing depressive symptoms, anxiety, stress, and psychological distress and improving QoL.

## Introduction

### Background

Acceptance and commitment therapy (ACT) is an empirically supported transdiagnostic approach that involves mindfulness and acceptance processes and behavior change processes for valued living [1,2]. ACT aims to develop greater psychological flexibility, or the ability to face challenging experiences in an open manner and change one’s behaviors to participate in valued activities, rather than avoiding uncomfortable or painful experiences, emotions, and thoughts [1,2]. ACT is based on a psychological flexibility model involving six processes [2]: (1) acceptance (ie, being open to unwanted thoughts and emotions as they are), (2) cognitive defusion (ie, stepping back from unhelpful thoughts and emotions to reduce their dominance over behaviors), (3) being present (ie, maintaining contact with the present moment), (4) observing self (ie, flexible self-conceptualization and perspective taking), (5) values (ie, clarifying personal values), and (6) committed action (ie, establishing patterns of behaviors for valued living) [2].

A growing body of evidence shows that ACT can reduce depressive symptoms, anxiety, stress, and psychological distress and improve quality of life (QoL) in various populations [3,4]. For example, previous meta-analyses found that ACT had small to medium effects on reducing depressive symptoms and anxiety and improving QoL in family caregivers [4] and a medium effect on reducing depressive symptoms in people diagnosed with depression [3]. A majority of previous meta-analyses regarding ACT were limited to specific populations, such as people with chronic pain [5] and people with psychosis [6], which led to a small number of included studies for meta-analysis. In addition, subgroup analyses were not conducted according to the delivery method (eg, face-to-face ACT vs internet-based ACT [iACT]) in the previous meta-analyses because of the limited number of studies included for ACT [4,7].

Internet-based psychological interventions are easy to access and inexpensive; therefore, it is important to determine whether iACT is an effective alternative option for reducing depressive symptoms, anxiety, stress, and overall psychological distress and improving QoL [8]. Brown et al [9] conducted a meta-analysis to measure the effects of iACT on outcomes related to mental health and well-being in any population and found a small effect of iACT on depressive symptoms only. This could be because of the limited number of included studies for each outcome at that time, which included 10 studies for depressive symptoms, 7 studies for anxiety, and 8 studies for QoL. More recently, Thompson et al [10] conducted a meta-analysis to measure the effects of iACT on depression, anxiety, and QoL and found small effects of iACT on all these outcomes. However, stress and psychological distress were not included in the meta-analysis by Thompson et al [10]. It is also possible that more studies have been published since the search by Thompson et al [10], which was conducted in June 2019. More importantly, subgroup analyses for each outcome were not conducted according to the type of control group in any of the previous meta-analyses to determine whether the effects of iACT differed compared with active control groups provided with other comparable interventions and passive control groups provided with no intervention. In addition, other subgroup analyses (eg, subgroup analyses according to the use of therapist guidance in iACT) may be possible and may provide useful information.

### Objectives

This systematic review and meta-analysis aimed to assess the efficacy of iACT for depressive symptoms, anxiety, stress, overall psychological distress, and QoL in any population, with subgroup analyses according to the type of control group and other possible subgroup analyses depending on the characteristics of the included studies, when applicable.

## Methods

### Overview

This study followed the PRISMA (Preferred Reporting Items for Systematic Reviews and Meta-Analyses) guideline [11] and the *Cochrane Handbook for Systematic Reviews of Interventions* (version 5.1.0) [12]. However, this study was not preregistered.

### Inclusion and Exclusion Criteria

Studies were selected based on the following inclusion criteria: (1) the study must be a randomized controlled trial (RCT); (2) ACT must be mainly delivered on the web (ie, iACT); (3) the study must have pre- and posttest results in measures of depressive symptoms, anxiety, stress, psychological distress, or QoL; (4) the study must compare iACT with a non-ACT comparison or a control condition; and (5) the study must be written in English. Studies were excluded if they compared only different delivery modes among the ACT groups (eg, ACT delivered on the web vs ACT delivered in person) without any other comparison or a control condition.

### Search Strategy

A comprehensive search was conducted using 4 electronic databases from the date of inception of each database to June 5, 2021: PubMed (1966-2021), CINAHL (1981-2021), PsycINFO (1935-2021), and SCOPUS (1966-2021). Key search terms were combined using keywords for iACT to identify the relevant literature. To broaden the database search, keywords for the outcomes were not entered as search terms. The search terms used in PubMed were as follows: (“acceptance and commitment therapy”[tiab] OR “Acceptance and Commitment Therapy”[MeSH]) AND (online[tiab] OR e-health[tiab] OR Internet*[tiab] OR web[tiab] OR webs[tiab] OR “web-based”[tiab] OR “web-delivered”[tiab] OR computer*[tiab] OR app[tiab] OR apps[tiab] OR mobile[tiab] OR technolog*[tiab] OR “Computers”[Mesh] OR “Internet-Based Intervention”[MeSH] OR “Telemedicine”[MeSH] OR “Distance Counseling”[MeSH] OR “Mobile Applications”[MeSH]). The full search strategies for all databases can be found in Multimedia Appendix 1. Articles were also manually searched using the reference lists of the identified articles and the related article features in the databases.

### Data Extraction and Quality Assessment

The characteristics of the included RCTs (eg, sample size, characteristics of participants, brief description of intervention and control groups, outcome measures, and results) were extracted into a table. Data regarding the means, SD, and sample sizes of each group were entered into an Excel (Microsoft) file. The risk of bias in the included RCTs was assessed using the Cochrane Collaboration risk-of-bias tool [12]. The domains in the tool include random sequence generation, allocation concealment, blinding of participants and personnel, blinding of outcome assessment, incomplete outcome data, and selective reporting. The risk of bias in each of the domains was judged as *low risk* of bias, *high risk* of bias, or *unclear risk* of bias according to the criteria provided in the Cochrane Collaboration handbook [12]. Summary assessments of the risk of bias within a study and across studies were also determined based on the handbook’s criteria [12]. One author completed the process for data extraction and quality assessment.

### Meta-analysis

Means, SDs, and sample sizes of the intervention and control groups in the included studies were entered into RevMan (version 5.4; Cochrane Collaboration) for meta-analysis and pooled for each outcome at the immediate posttest and at follow-up. The *I*^²^ statistic was used to measure the statistical heterogeneity across studies, and an *I^2^*>60% was interpreted as substantial heterogeneity [12]. The decision to use either a random effects model or a fixed effects model with the inverse variance method was determined by the *I^2^* statistic values for each outcome, that is, a random effects model was used when the *I^2^* statistic for each variable was >60%; otherwise, a fixed effects model was used. A significance level (*P* value) of .05 was used. The standardized mean difference (SMD) with 95% CIs was used as a summary statistic for the size of the intervention effect to account for outcomes measured using different assessment tools [12]. SMDs <0.4 indicate a small effect, SMDs between 0.4 and 0.7 indicate a medium effect, and SMDs >0.7 indicate a large effect [12]. Subgroup analyses for each outcome were conducted according to the type of control group, if applicable, to see whether the effects of iACT differed compared with active control groups provided with other comparable interventions and passive control groups provided with no intervention (ie, treatment-as-usual control groups and waitlist control groups).

## Results

### Selection of Studies

**Figure 1:**
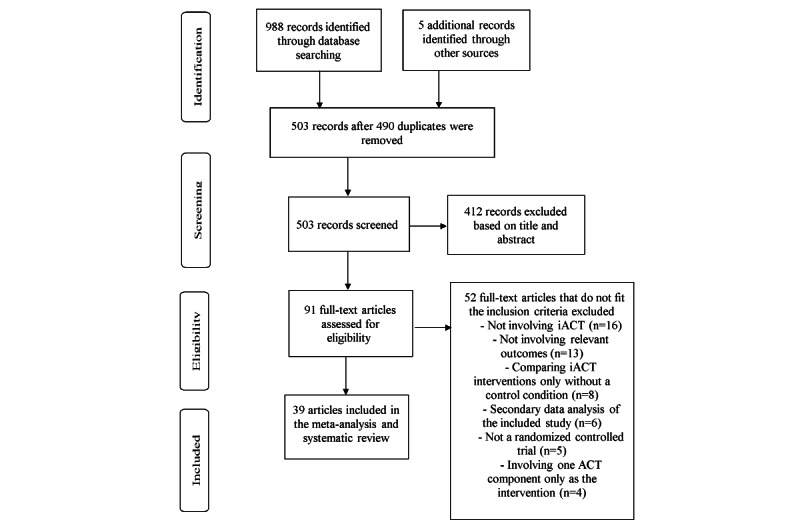
PRISMA (Preferred Reporting Items for Systematic Reviews and Meta-Analyses) flow diagram of study selection process. ACT: acceptance and commitment therapy; iACT: internet-based acceptance and commitment therapy.

Figure 1 illustrates the study selection process. A total of 988 articles were identified through database searching, and 5 additional articles were identified through manual searching. After removing 490 duplicates, 503 articles were screened based on title and abstract screening. A total of 412 articles were excluded based on title and abstract screening, and 91 articles were assessed for eligibility by reading the full text. A total of 52 articles were excluded after reading the full text because of the following reasons: not involving iACT (16 studies), not involving relevant outcomes (13 studies), comparing different delivery methods of iACT interventions without a control condition (8 studies), secondary data analysis of the included study (6 studies), not an RCT (5 studies), and involving 1 ACT component only (4 studies). A total of 39 articles met the eligibility criteria [13-51].

### Characteristics of the Included Studies

The main characteristics of the included 39 RCTs are summarized in Multimedia Appendix 2 [13-51]. The average number of ACT modules (sessions) in the included studies was 6.7 (SD 2.5), ranging from 2 modules to 13 modules. ACT was delivered on the web with therapist guidance in 32 studies (eg, via videoconferencing, phone calls, written feedback, and a mobile app) and without therapist guidance in 7 studies [13-19]. A total of 8 studies used a blended ACT program involving both iACT and in-person ACT sessions [20-27]. In addition, 2 studies involved videoconferencing ACT [28,29]. The remaining 29 RCTs involved web-based ACT modules. Of the 39 RCTs, 10 studies involved active control groups, including web-based discussion forums [35,37,41], web-based expressive writing [32,51], web-based mental health education [17], a web-based smoking cessation intervention [14], web-based cognitive behavioral therapy (CBT) [13], in-person CBT [27], and in-person documentary discussions [25]. Moreover, 18 studies directly targeted people with depressive symptoms [14,21,23-25,27,30-40]. Additional subgroup analyses were conducted because studies could be categorized according to the following 3 characteristics: use of therapist guidance, delivery modes (ie, web-based ACT modules, iACT accompanied by in-person ACT sessions, and videoconferencing ACT), and use of targeted participants (eg, studies that directly targeted participants with depressive symptoms vs studies that involved participants regardless of the depressive symptoms used for subgroup analysis of depressive symptoms).

The average sample size of participants in the included RCTs was 139 (SD 185), ranging from 24 to 1162. The mean age of the participants was 40.1 (SD 13.3) years, ranging from 15.3 to 63.1 years, and the average percentage of female participants was 69.14% (3791/5414; SD 19.5%), ranging from 0% to 98.5%. The included RCTs were conducted in Sweden (10 studies), the United States (8 studies), the Netherlands (6 studies), Finland (5 studies), the United Kingdom (2 studies), Australia (2 studies), Canada (1 study), Belgium (1 study), Ireland (1 study), France (1 study), Denmark (1 study), and Germany (1 study). Of the 39 included studies, 30 (77%) were published between 2016 and 2021, and the remaining 9 (23%) were published between 2012 and 2015.

The following section describes the results of the meta-analyses regarding the efficacy of iACT for depressive symptoms, anxiety, stress, psychological distress, and QoL at the immediate posttest and follow-up. Subgroup analyses for each outcome were performed according to the type of control groups (ie, iACT vs active control groups and iACT vs passive control groups) when applicable.

### Effects of iACT on Reducing Depressive Symptoms

**Figure 2:**
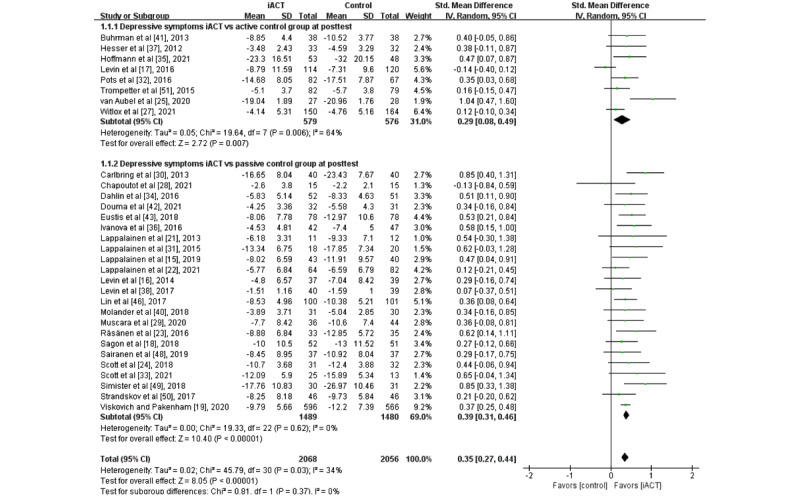
Forest plots showing effects of internet-based acceptance and commitment therapy on depressive symptoms at the immediate posttest. iACT: internet-based acceptance and commitment therapy [15-19,21-25,27-38,40-43,46,48-51].

A meta-analysis of 31 RCTs (N=4124 participants) showed that iACT had a small effect on reducing depressive symptoms at the immediate posttest compared with control groups overall (SMD 0.35, 95% CI 0.27-0.44; Figure 2). There was no statistically significant subgroup difference at the immediate posttest (*χ*^2^_1_=0.8; *P*=.37), indicating that the effects of the 2 subgroups (ie, subgroup 1: iACT vs active control groups and subgroup 2: iACT vs passive control groups) at the immediate posttest were not statistically different from one another. Small effects of iACT on depressive symptoms were found at the immediate posttest regardless of the control group conditions in 8 studies (N=1155 participants) that compared iACT with active control groups (SMD 0.29, 95% CI 0.08-0.49) and 23 studies (N=2969) that compared iACT with passive control conditions (SMD 0.39, 95% CI 0.31-0.46).

**Figure 3:**
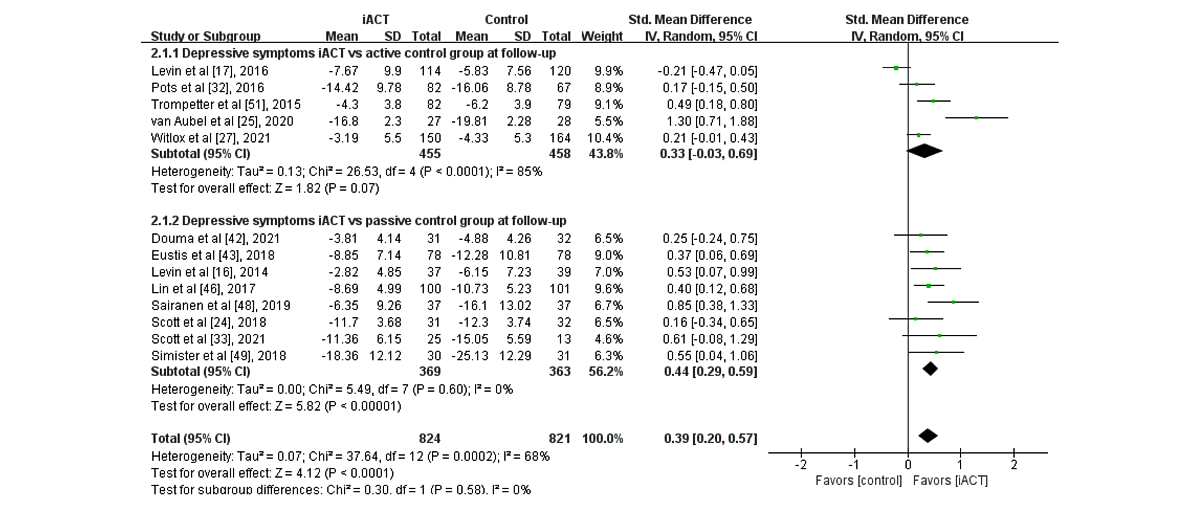
Forest plots showing effects of internet-based acceptance and commitment therapy on depressive symptoms at follow-up. iACT: internet-based acceptance and commitment therapy [16,17,24,25,27,32,33,42,43,46,48,49,51].

A meta-analysis of 13 RCTs with follow-up data (N=1645 participants) revealed that iACT had a small effect on reducing depressive symptoms at follow-up compared with control groups overall (SMD 0.39, 95% CI 0.20-0.57; Figure 3). There was no statistically significant subgroup difference at follow-up (*χ*^2^_1_=0.3; *P*=.58), indicating that the effects of the 2 subgroups (ie, subgroup 1: iACT vs active control groups and subgroup 2: iACT vs passive control groups) at follow-up were not statistically different from one another. The iACT had a medium effect compared with passive control groups at follow-up (8 studies that involved 732 participants, SMD 0.44, 95% CI 0.29-0.59), but iACT was not significantly different from active control groups (5 studies that involved 913 participants, SMD 0.33, 95% CI −0.03 to 0.69).

### Effects of iACT on Reducing Anxiety

**Figure 4:**
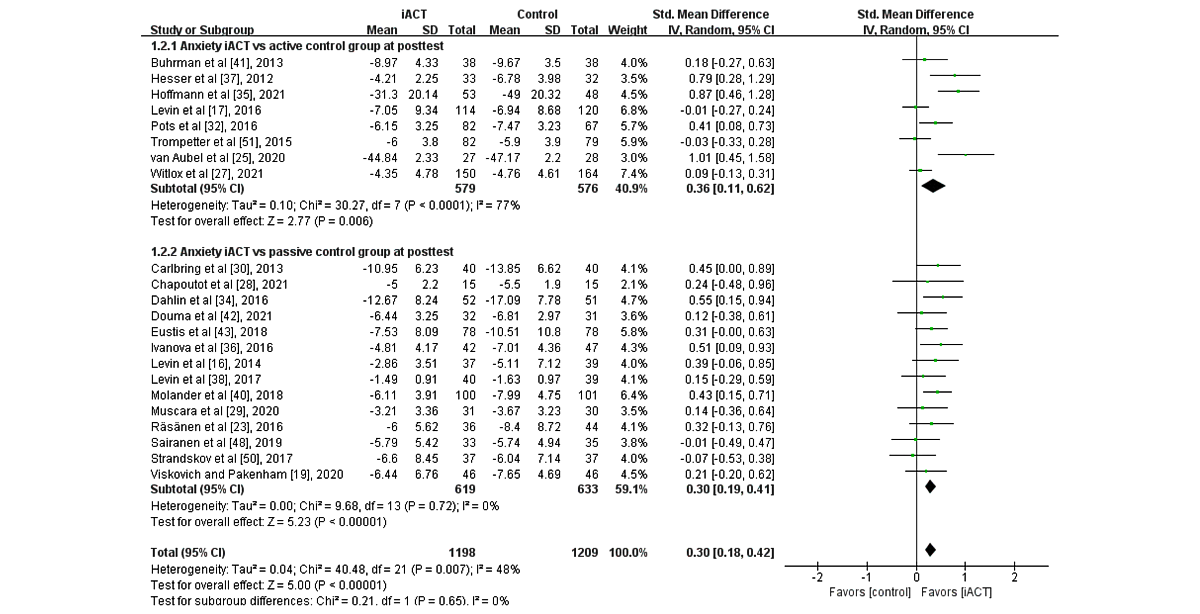
Forest plots showing effects of internet-based acceptance and commitment therapy on anxiety at the immediate posttest. iACT: internet-based acceptance and commitment therapy [16,17,19,23,25,27-30,32,34-38,40-43,48,50,51].

A meta-analysis of 22 RCTs (N=2407 participants) found that iACT had a small effect on reducing anxiety at the immediate posttest compared with control groups overall (SMD 0.30, 95% CI 0.18-0.42; Figure 4). There was no statistically significant subgroup difference at the immediate posttest (*χ*^2^_1_=0.2; *P*=.65), indicating that the effects of the 2 subgroups (ie, subgroup 1: iACT vs active control groups and subgroup 2: iACT vs passive control groups) at the immediate posttest were not statistically different from one another. Small effects of iACT on anxiety were found at the immediate posttest regardless of the control group conditions in 8 studies (N=1155 participants) that compared iACT with active control groups (SMD 0.36, 95% CI 0.11-0.62) and 14 studies (N=1251 participants) that compared iACT with passive control conditions (SMD 0.30, 95% CI 0.19-0.41).

**Figure 5:**
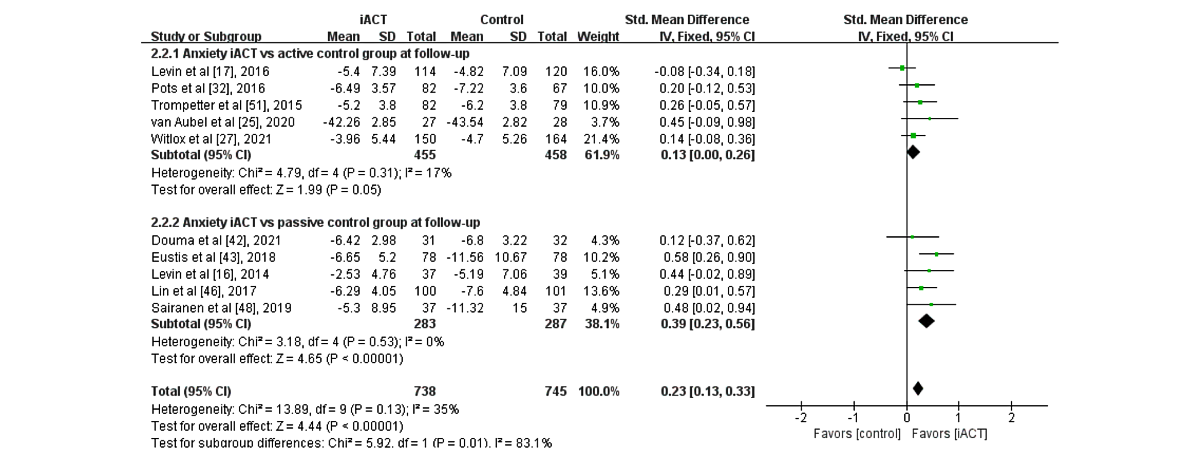
Forest plots showing effects of internet-based acceptance and commitment therapy on anxiety at follow-up. iACT: internet-based acceptance and commitment therapy [16,17,25,27,32,42,43,46,48,51].

A meta-analysis of 10 RCTs with follow-up data (N=1483 participants) showed that iACT had a small effect on reducing anxiety at follow-up compared with control groups overall (SMD 0.23, 95% CI 0.13-0.33; Figure 5). There was a statistically significant subgroup difference at follow-up (*χ*^2^_1_=5.9; *P*=.01), indicating that the effects of the 2 subgroups (ie, subgroup 1: iACT vs active control groups and subgroup 2: iACT vs passive control groups) at follow-up were statistically different from one another. At follow-up, iACT had small effects compared with both passive control groups (5 studies that involved 570 participants, SMD 0.39, 95% CI 0.23-0.56) and active control groups (5 studies that involved 913 participants, SMD 0.13, 95% CI 0.00-0.26), but the effect size of iACT was larger compared with passive control groups.

### Effects of iACT on Reducing Stress

**Figure 6:**
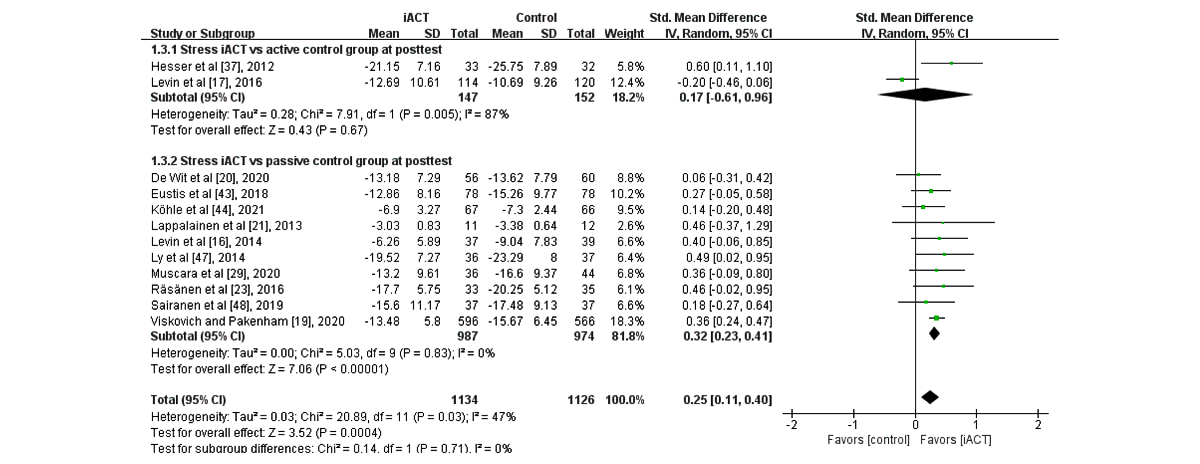
Forest plots showing effects of internet-based acceptance and commitment therapy on stress at the immediate posttest. iACT: internet-based acceptance and commitment therapy [16,17,19,20,21,23,29,37,43,44,47,48].

A meta-analysis of 12 RCTs (N=2260 participants) revealed that iACT had a small effect on reducing stress at the immediate posttest compared with control groups overall (SMD 0.25, 95% CI 0.11-0.40; Figure 6). There was no statistically significant subgroup difference at the immediate posttest (*χ*^2^_1_=0.1; *P*=.71), indicating that the effects of the 2 subgroups (ie, subgroup 1: iACT vs active control groups and subgroup 2: iACT vs passive control groups) at the immediate posttest were not statistically different from one another. The iACT had a small effect on reducing stress compared with passive control groups at the immediate posttest (10 studies that involved 1961 participants, SMD 0.32, 95% CI 0.25-0.43), but iACT was not significantly different from active control groups (2 studies that involved 299 participants, SMD 0.17, 95% CI −0.61 to 0.96).

**Figure 7:**
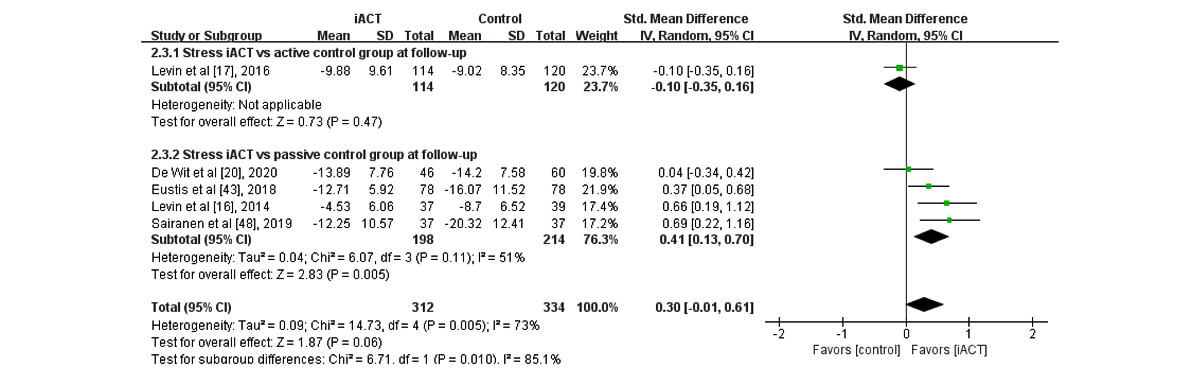
Forest plots showing effects of internet-based acceptance and commitment therapy on stress at follow-up. iACT: internet-based acceptance and commitment therapy [16,17,20,43,48].

A meta-analysis of 5 RCTs with follow-up data (N=646 participants) found that iACT did not differ from control groups in reducing stress at follow-up overall (SMD 0.30, 95% CI −0.01 to 0.61; Figure 7). There was a statistically significant subgroup difference at follow-up (*χ*^2^_1_=6.7; *P*=.01), indicating that the effects of the 2 subgroups (ie, subgroup 1: iACT vs active control groups and subgroup 2: iACT vs passive control groups) at follow-up were statistically different from one another. The iACT had a medium effect on reducing stress compared with passive control groups at follow-up (4 studies that involved 412 participants, SMD 0.41, 95% CI 0.13-0.70), but iACT was not significantly different from active control groups (1 study that involved 234 participants, SMD −0.10, 95% CI −0.35 to 0.16).

### Effects of iACT on Reducing Psychological Distress

**Figure 8:**
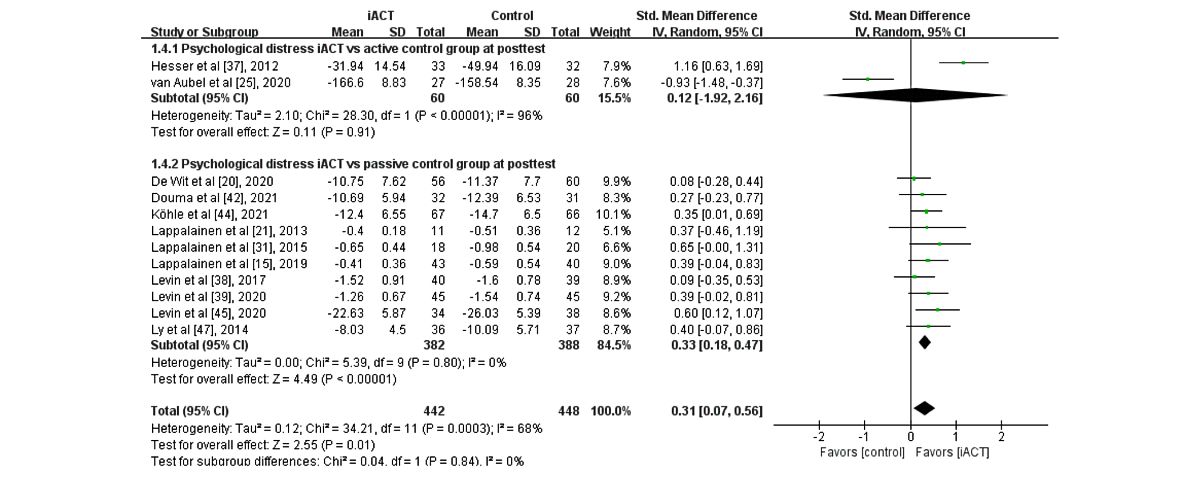
Forest plots showing effects of internet-based acceptance and commitment therapy on psychological distress at the immediate posttest. iACT: internet-based acceptance and commitment therapy [15,20,21,25,31,37-39,42,44,45,47].

A meta-analysis of 12 RCTs (N=890 participants) found that iACT had a small effect on reducing psychological distress at the immediate posttest compared with control groups overall (SMD 0.31, 95% CI 0.07-0.56; Figure 8). There was no significant subgroup difference at the immediate posttest (*χ*^2^_1_=0.0; *P*=.84), indicating that the effects of the 2 subgroups (ie, subgroup 1: iACT vs active control groups and subgroup 2: iACT vs passive control groups) at the immediate posttest were not statistically different from one another. The iACT had a small effect on reducing psychological distress compared with passive control groups at the immediate posttest (10 studies that involved 770 participants, SMD 0.33, 95% CI 0.18-0.47), but iACT was not significantly different from active control groups (2 studies that involved 120 participants, SMD 0.12, 95% CI −1.92 to 2.16).

**Figure 9:**
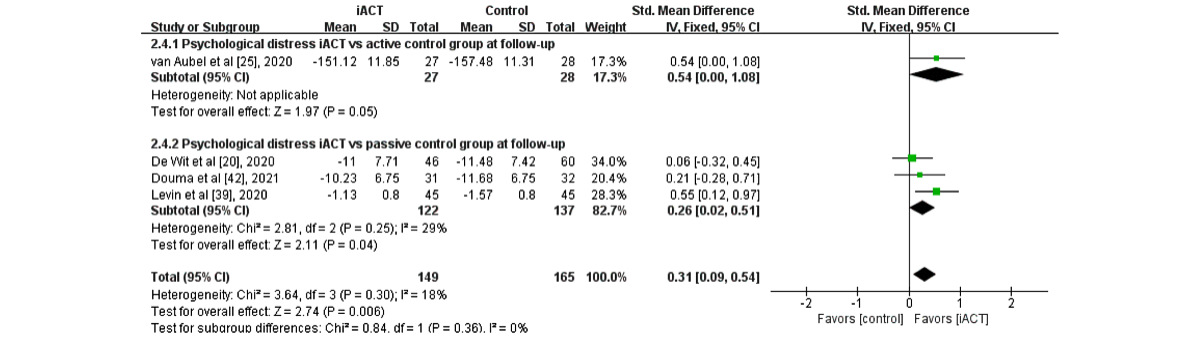
Forest plots showing effects of internet-based acceptance and commitment therapy on psychological distress at follow-up. iACT: internet-based acceptance and commitment therapy [20,25,39,42].

A meta-analysis of 4 RCTs with follow-up data (N=314 participants) revealed that iACT had a small effect on reducing psychological distress at follow-up compared with control groups overall (SMD 0.31, 95% CI 0.09-0.54; Figure 9). There was no significant subgroup difference at follow-up (*χ*^2^_1_=0.8; *P*=.36), indicating that the effects of the 2 subgroups (ie, subgroup 1: iACT vs active control groups and subgroup 2: iACT vs passive control groups) at follow-up were statistically different from one another. The iACT had a medium effect on psychological distress compared with an active control group at follow-up (1 study that involved 55 participants, SMD 0.54, 95% CI 0.00-1.08), whereas the iACT had a small effect compared with passive control groups at follow-up (3 studies that involved 259 participants, SMD 0.26, 95% CI 0.02-0.51).

### Effects of iACT on Improving QoL

**Figure 10:**
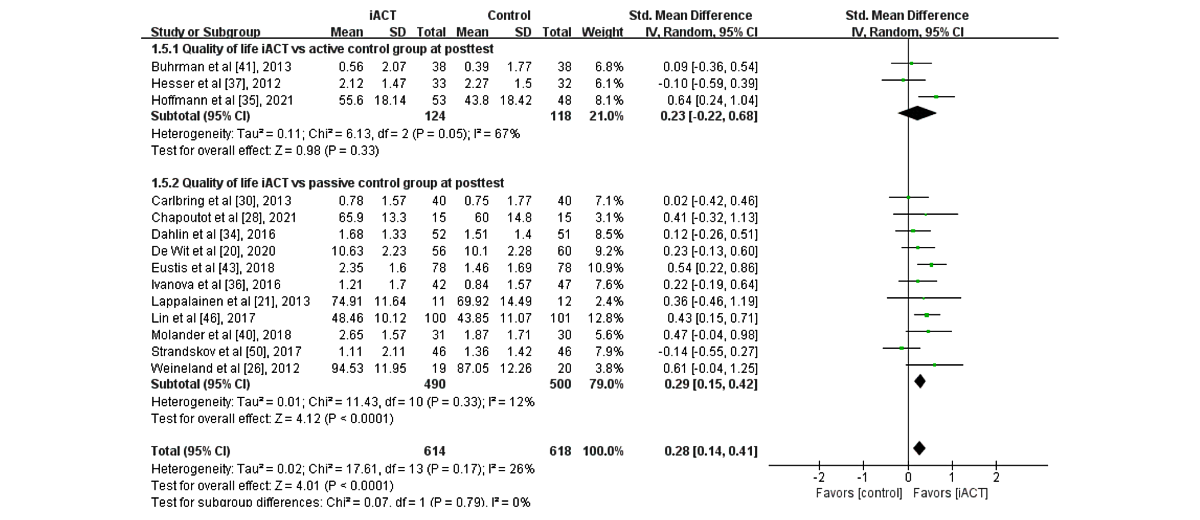
Forest plots showing effects of internet-based acceptance and commitment therapy on quality of life at the immediate posttest. iACT: internet-based acceptance and commitment therapy [20,21,26,28,30,34-37,40,41,43,46,50].

A meta-analysis of 14 RCTs (N=1232 participants) showed that iACT had a small effect on improving QoL at the immediate posttest compared with control groups overall (SMD 0.28, 95% CI 0.14-0.41; Figure 10). There was no statistically significant subgroup difference at the immediate posttest (*χ*^2^_1_=0.1; *P*=.79), indicating that the effects of the 2 subgroups (ie, subgroup 1: iACT vs active control groups and subgroup 2: iACT vs passive control groups) at the immediate posttest were not statistically different from one another. The iACT had a small effect on improving QoL compared with passive control groups at the immediate posttest (11 studies that involved 990 participants, SMD 0.29, 95% CI 0.15-0.42), but iACT was not significantly different from active control groups (3 studies that involved 242 participants, SMD 0.23, 95% CI −0.22 to 0.68).

**Figure 11:**
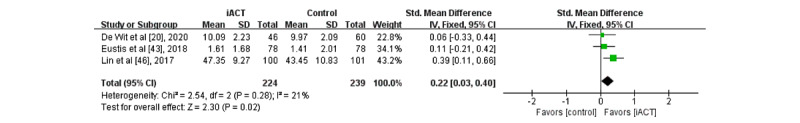
Forest plots showing effects of internet-based acceptance and commitment therapy on quality of life at follow-up. iACT: internet-based acceptance and commitment therapy.

All 3 studies with follow-up data (N=463 participants) compared iACT with passive control groups only, so no subgroup analysis at follow-up was conducted. The iACT had a small effect on improving QoL compared with passive control groups at follow-up (SMD 0.22, 95% CI 0.03-0.40; Figure 11).

### Subgroup Analyses According to the Use of Therapist Guidance

Subgroup analyses showed small effects of iACT with therapist guidance on depressive symptoms at the immediate posttest (26 studies that involved 2466 participants, SMD 0.38, 95% CI 0.29-0.48), anxiety at the immediate posttest (20 studies that involved 2097 participants, SMD 0.30, 95% CI 0.21-0.38) and at follow-up (8 studies that involved 1173 participants, SMD 0.28, 95% CI 0.17-0.40), stress at the immediate posttest (9 studies that involved 788 participants, SMD 0.28, 95% CI 0.14-0.42) and at follow-up (3 studies that involved 336 participants, SMD 0.35, 95% CI 0.01-0.68), and psychological distress at the immediate posttest (11 studies that involved 807 participants, SMD 0.31, 95% CI 0.04-0.57) compared with control groups. A subgroup analysis found a medium effect of iACT with therapist guidance on depressive symptoms at follow-up (11 studies that involved 1335 participants, SMD 0.43, 95% CI 0.27-0.59). However, subgroup analyses revealed small effects of iACT without therapist guidance on depressive symptoms (5 studies that involved 1658 participants, SMD 0.24, 95% CI 0.00-0.48) and anxiety (3 studies that involved 1472 participants, SMD 0.18, 95% CI 0.08-0.28) at the immediate posttest only, with smaller effect sizes than those for iACT with therapist guidance. The iACT without therapist guidance was not significantly different from the control groups in depressive symptoms at follow-up (2 studies that involved 310 participants, SMD 0.14, 95% CI −0.59 to 0.86), anxiety at follow-up (2 studies that involved 310 participants, SMD 0.14, 95% CI −0.36 to 0.64), stress at the immediate posttest (4 studies that involved 1514 participants, SMD 0.08, 95% CI −0.29 to 0.46) and at follow-up (2 studies that involved 310 participants, SMD 0.25, 95% CI −0.48 to 0.99), and psychological distress at the immediate posttest (2 studies that involved 125 participants, SMD 0.09, 95% CI −0.56 to 0.74). There was no statistically significant subgroup difference in all the outcomes, indicating no statistically significant difference among studies according to the use of therapist guidance. Forest plots of these subgroup analyses are illustrated in Figures S1-S7 in Multimedia Appendix 3 [13,15-25,27-51].

### Subgroup Analyses According to Delivery Mode

Subgroup analyses showed small effects of web-based ACT on depressive symptoms at the immediate posttest (23 studies that involved 3345 participants, SMD 0.35, 95% CI 0.28-0.42) and at follow-up (10 studies that involved 1213 participants, SMD 0.36, 95% CI 0.16-0.57), anxiety at the immediate posttest (18 studies that involved 3022 participants, SMD 0.29, 95% CI 0.18-0.40) and at follow-up (8 studies that involved 1114 participants, SMD 0.25, 95% CI 0.13-0.37), stress at the immediate posttest (9 studies that involved 2015 participants, SMD 0.21, 95% CI 0.03-0.40), and QoL at the immediate posttest (10 studies that involved 1024 participants, SMD 0.28, 95% CI 0.15-0.40) and at follow-up (2 studies that involved 357 participants, SMD 0.26, 95% CI 0.05-0.47) and medium effects of web-based ACT on psychological distress at the immediate posttest (10 studies that involved 738 participants, SMD 0.40, 95% CI 0.20-0.60) and at follow-up (2 studies that involved 153 participants, SMD 0.41, 95% CI 0.08-0.73) compared with control groups. Subgroup analyses revealed small effects of iACT accompanied by in-person ACT sessions on depressive symptoms (6 studies that involved 669 participants, SMD 0.28, 95% CI 0.13-0.43) and QoL (3 studies that involved 178 participants, SMD 0.33, 95% CI 0.03-0.63) at the immediate posttest only compared with control groups, findings that may be because of a smaller number of studies included for these subgroup analyses. Few studies compared videoconferencing ACT with control groups (ie, 2 studies for depressive symptoms and anxiety), and these subgroup analyses showed no statistically significant difference of videoconferencing ACT from control groups in these outcomes. There was no statistically significant subgroup difference in all the outcomes (*P*>.05), indicating no statistically significant difference among the studies according to delivery mode. Forest plots of these subgroup analyses are illustrated in Figures S8-S17 in Multimedia Appendix 4 [13,15-51].

### Subgroup Analyses According to the Targeted Participants

Subgroup analyses found medium effects of iACT on depressive symptoms (5 studies that involved 360 participants, SMD 0.62, 95% CI 0.40-0.83]) and anxiety (4 studies that involved 607 participants, SMD 0.48, 95% CI 0.11-0.85) at the immediate posttest compared with control groups when studies directly targeted participants with depressive symptoms and anxiety, respectively. In contrast, subgroup analyses showed small effects of iACT on depressive symptoms (26 studies that involved 3764 participants, SMD 0.31, 95% CI 0.25-0.37) and anxiety (19 studies that involved 2962 participants, SMD 0.24, 95% CI 0.14-0.35) at the immediate posttest compared with control groups when studies did not involve targeted participants for each of the outcomes (eg, studies that involved participants regardless of depressive symptoms at baseline). Subgroup analyses found small effects of iACT on stress at the immediate posttest (11 studies that involved 2199 participants, SMD 0.26, 95% CI 0.17-0.34) and on depressive symptoms (10 studies that involved 1403 participants, SMD 0.33, 95% CI 0.15-0.52) and anxiety (9 studies that involved 1169 participants, SMD 0.26, 95% CI 0.14-0.37) at follow-up compared with control groups when studies did not involve targeted participants for each of the outcomes. However, there was no statistically significant difference of iACT from control groups in stress and psychological distress at the immediate posttest and in depressive symptoms and anxiety at follow-up when studies directly targeted participants for each of the outcomes, findings that may be because of a smaller number of studies included for these subgroup analyses (eg, 2 studies for stress). There was no statistically significant subgroup difference in all the outcomes (*P*>.05), except for depressive symptoms at the immediate posttest (*P*=.007). These findings indicate that there was no statistically significant difference among studies according to the use of targeted participants in all the outcomes, except for depressive symptoms at the immediate posttest, for which a statistically significant, larger effect of iACT was found when studies targeted people with depressive symptoms (ie, SMD 0.62 vs SMD 0.31). Forest plots of these subgroup analyses are illustrated in Figures S18-S24 in Multimedia Appendix 5 [13,15-25,27-51].

### Risk of Bias of the Included Studies

Of the 39 included studies, 20 (51%) had an unclear risk of bias, 14 (36%) had a low risk of bias, and 5 (13%) had a high risk of bias overall (Multimedia Appendix 6 [13-51]). A domain for blinding of participants and personnel was not considered as the key domain for the overall risk of bias within a study because studies that involved passive control conditions were less able to conceal the group allocation from participants. The overall risk of bias across 39 studies was interpreted as unclear because most information was from studies with an unclear risk of bias [12].

## Discussion

### Principal Findings

This systematic review with meta-analysis identified 39 studies that assessed the efficacy of iACT for depressive symptoms, anxiety, stress, psychological distress, and QoL. This study found that iACT had small effects on reducing depressive symptoms, anxiety, stress, and psychological distress and improving QoL at the immediate posttest and follow-up. There was no significant effect of iACT on stress at follow-up.

One of the previous meta-analyses found that iACT had a small effect on depressive symptoms and nonsignificant effects on anxiety and QoL; it included 10 studies involving depressive symptoms, 7 studies involving anxiety, and 8 studies involving QoL [9]. As more studies on iACT (ie, 30 studies on iACT) have been conducted since 2016, this study’s meta-analyses included 31 studies on depressive symptoms, 22 studies on anxiety, and 14 studies on QoL. This may explain why, contrary to Brown et al [9], this study found small effects of iACT on anxiety and QoL in addition to depressive symptoms with the increased statistical power from a larger total sample size pooled from more studies.

Another prior meta-analysis [10] found small effects of iACT on depressive symptoms (25 studies at posttest) and anxiety (20 studies at posttest), so this study confirms the previous meta-analysis with more studies added (ie, 6 more studies for depressive symptoms and 2 more studies for anxiety). More importantly, this study found small effects of iACT on stress and psychological distress, which were not included in the meta-analyses by Brown et al [9] and Thompson et al [10]. Such positive findings are supported by previous studies that suggested negative relationships of the ACT processes, such as mindfulness and acceptance, with depressive symptoms, anxiety, stress, and psychological distress, and positive relationships with QoL [52,53]. ACT promoting acceptance, mindfulness, and committed action to living in alignment with values may improve QoL and decrease psychological distress by helping those who receive the therapy better manage uncomfortable or negative emotions, thoughts, and experiences; accept them without judgment; and move forward to valued living [54,55]. The findings of this study contribute to the body of evidence regarding the effects of iACT on depressive symptoms, anxiety, stress, psychological distress, and QoL and may be applicable in any population as ACT is a transdiagnostic approach [1].

Subgroup analyses for each outcome were conducted in this study according to the type of control groups but were not conducted in prior meta-analyses [9,10]. Subgroup analyses showed small to medium effects of iACT on all the outcomes at the immediate posttest and follow-up compared with passive control groups. In contrast, subgroup analyses that compared iACT with active control groups (eg, CBT and mental health education) found no differences between groups on stress, psychological distress, and QoL at the immediate posttest or on depressive symptoms, anxiety, and stress at follow-up.

However, it should be noted that relatively fewer studies have compared the effects of iACT with active control conditions. There were 2 to 5 times more studies that compared iACT with passive control conditions than those that compared iACT with active control conditions in all the outcomes, except for anxiety at follow-up. There were fewer studies that compared iACT with active control conditions, especially for stress and psychological distress at the immediate posttest and follow-up. More importantly, active control conditions varied among the included studies, including web-based discussion forums [35,37,41], web-based expressive writing [32,51], web-based mental health education [17], a web-based smoking cessation intervention [14], web-based CBT [13], in-person CBT [27], and in-person documentary discussions [25]. Such differences in the active control conditions might explain the higher statistical heterogeneity when comparing iACT with active control groups than heterogeneity when comparing iACT with passive control groups. Overall, these findings suggest a need for more studies comparing iACT with active control conditions enough to assess whether iACT is comparable or superior to each of the other evidence-based interventions in decreasing depressive symptoms, anxiety, stress, and psychological distress and improving QoL in future meta-analyses.

This study conducted subgroup analyses according to the use of therapist guidance, delivery modes, and the use of targeted participants and found no statistically significant subgroup differences among studies according to these 3 characteristics in all the outcomes, except for the subgroup difference among studies according to the use of targeted participants for depressive symptoms at the immediate posttest (ie, a statistically significant, larger effect of iACT when studies targeted people with depressive symptoms). However, more studies are needed to confirm these findings, especially in subgroup analyses according to delivery modes and targeted participants, as there were only a few studies that involved delivery modes other than web-based ACT modules or that involved directly targeted participants (eg, only 2 studies that involved people with stress).

### Limitations

This review had some limitations. The Bonferroni correction method might be needed to adjust the results of multiple comparisons to reduce the false positive error rate (ie, type I errors) in a meta-analysis [56]. As 10 comparisons were made in this meta-analysis, the level of significance (*P* value) after Bonferroni correction was .005 (0.05/10). With the adjusted level of significance, most results remained the same, except for psychological distress at posttest and follow-up and QoL at follow-up. As RevMan does not allow users to choose the levels of significance other than 0.1, 0.05, or 0.01, and the values in the forest plots, including CIs, cannot be changed accordingly, we could not use the adjusted level of significance (ie, *P*=.005) in this meta-analysis. A total of 4 electronic databases were searched; therefore, some relevant articles could have been missed if they were published only in other databases. Only studies written in English were searched and included in this review, which could create a publication bias. One author with extensive experience in comprehensive literature reviews and expertise in ACT searched the literature; therefore, this review did not include 2 independent reviewers in the search process. A recent methodological systematic review found that single screening for study selection in systematic reviews conducted by an experienced reviewer had no impact on the findings of the meta-analysis [57]. The overall risk of bias across the included RCTs was interpreted as unclear, indicating the need for high-quality studies to better determine the efficacy of iACT for psychological distress and QoL. This study did not aim to compare iACT with face-to-face ACT because the focus was on comparing iACT with non-ACT control conditions. Further meta-analyses may consider comparing iACT with face-to-face ACT to determine whether iACT is comparable with face-to-face ACT when more studies are available. Further meta-analyses may consider examining whether the effects of blended ACT, which involves iACT and face-to-face ACT, differ from iACT only or face-to-face ACT only.

## Appendix

Multimedia Appendix 1 Search terms used in database searches.

Multimedia Appendix 2 Characteristics of the included studies.

Multimedia Appendix 3 Forest plots subgroup analyses according to the use of therapist guidance (Figures S1-S7).

Multimedia Appendix 4 Forest plots subgroup analyses according to delivery mode (Figures S8-S17).

Multimedia Appendix 5 Forest plots subgroup analyses according to the targeted participants (Figures S18-S24).

Multimedia Appendix 6 Forest plots showing effects of internet-based acceptance and commitment therapy on quality of life at follow-up. iACT: internet-based acceptance and commitment therapy [20,43,46].

## Abbreviations

ACT: acceptance and commitment therapy
CBT: cognitive behavioral therapy
iACT: internet-based acceptance and commitment therapy
PRISMA: Preferred Reporting Items for Systematic Reviews and Meta-Analyses
QoL: quality of life
RCT: randomized controlled trial
SMD: standardized mean difference
