# Supplementary material for: Efficacy of Internet-Based Acceptance and Commitment Therapy for Depressive Symptoms, Anxiety, Stress, Psychological Distress, and Quality of Life: Systematic Review and Meta-analysis
Source: J Med Internet Res. 2022 Dec 9;24(12):e39727. doi: 10.2196/39727 (PMC9789494; doi:10.2196/39727)
Supplement: Multimedia Appendix 1 [file jmir_v24i12e39727_app1.pdf]

Search Terms Used in Database Searches

Database	Search Terms
PubMed	(“acceptance and commitment therapy”[tiab] OR “Acceptance and Commitment Therapy”[MeSH]) AND (online[tiab] OR e-health[tiab] OR Internet*[tiab] OR web[tiab] OR webs[tiab] OR “web-based”[tiab] OR “web-delivered”[tiab] OR computer*[tiab] OR app[tiab] OR apps[tiab] OR mobile[tiab] OR technolog*[tiab] OR “Computers”[Mesh] OR “Internet-Based Intervention”[MeSH] OR “Telemedicine”[MeSH] OR “Distance Counseling”[MeSH] OR “Mobile Applications”[Mesh])
CINAHL	(TI "acceptance and commitment therapy" OR AB "acceptance and commitment therapy") AND (TI "online" OR AB "online" OR TI "e-health" OR AB "e-health" OR TI "internet*" OR AB "internet*" OR TI "web*" OR AB "web*" OR TI "computer*" OR AB "computer*" OR TI "app" OR AB "app" OR TI "apps" OR AB "apps" OR TI "mobile" OR AB "mobile" OR TI "technolog*" OR AB "technolog*")
PsycInfo®	(TIAB("acceptance and commitment therapy")) AND (TIAB(online OR e-health OR Internet* OR web* OR computer* OR app OR apps OR mobile OR technolog*))
Scopus	(TITLE-ABS-KEY ("acceptance and commitment therapy") AND TITLE-ABS-KEY (online OR e-health OR internet* OR web* OR computer* OR app OR apps OR mobile OR technolog*))
