# Supplementary material for: Efficacy of Internet-Based Acceptance and Commitment Therapy for Depressive Symptoms, Anxiety, Stress, Psychological Distress, and Quality of Life: Systematic Review and Meta-analysis
Source: J Med Internet Res. 2022 Dec 9;24(12):e39727. doi: 10.2196/39727 (PMC9789494; doi:10.2196/39727)
Supplement: Multimedia Appendix 2 [file jmir_v24i12e39727_app2.pdf]

Characteristics of the Included Studies

Reference; Country	Participants; Mean age; Female (%)	Descriptions of intervention and control groups	Relevant outcomes (measures); Data collection time points; Relevant results (between-group differences only)	Overall RoB
Barrett & Stewart (2021); Ireland	42 adults working within the social and healthcare professions; 37.1 years; 88%	- IG: A 2-week online ACT program (3 modules) for stress management, involving videos, brief informational questions that tracked understanding of and attention to the material, homework exercises, and ACT-based mindfulness exercises (*n* = 22)<br>- CG: A 2-week online CBT intervention for stress management, involving 3 modules (*n* = 20)	Stress (PSS) and psychological distress (GHQ-12); Pretest and posttest; No significant between-group differences in PSS and GHQ-12 over time	Unclear
Buhrman et al. (2013); Sweden	76 adults with functional impairment caused by chronic pain; 49.1 years; 59.2%	- IG: A 7-week guided internet-delivered ACT intervention for chronic pain, involving information about the ACT processes, assignments, relevant metaphors, and mindfulness exercises that could be downloaded as MP3 files + a text message as a reminder for homework assignments + 30-min phone calls at weeks 3 and 7 (*n* = 38)<br>- CG: A moderated online discussion forum for chronic pain, presenting weekly discussion topics by therapists and encouraging discussion (*n* = 38)	Depressive symptoms (HADS-D), anxiety (HADS-A), and QoL (QOLI); Pretest, posttest, and 6-month F/U; Significant improvements on HADS-D and HADS-A in IG compared to CG over time ($p < 0.05$)	Low
Carlbring et al. (2013); Sweden	80 adults diagnosed with major depression of mild to moderate severity; 44.4 years; 82.5%	- IG: 7 modules of internet-delivered behavioral activation combined with ACT (defusion, practice of acceptance and mindfulness, and values), involving online content (i.e., text, videos, and narrated animations) and homework assignments + a CD-ROM for mindfulness and acceptance exercises + written support and feedback by an internet therapist for 15 min per week (*n* = 40)<br>- CG: Waitlist control (*n* = 40)	Depressive symptoms (BDI-II), anxiety (BAI), and QoL (QOLI); Pretest, posttest, and 3-month F/U; Significant improvements on BDI-II and BAI in IG compared to CG over time ($p < 0.001$)	Unclear
Chapoutot et al. (2021); France	32 adults with chronic insomnia and hypnotic dependence; 48 years; 80%	- IG: Four 1-hr individual videoconference sessions delivered every 2 weeks by two psychologists trained in ACT and CBT for insomnia treatment + an ACT manual and audio recordings of mindfulness exercises (*n* = 16)<br>- CG: Waitlist control (*n* = 16)	Depressive symptoms (QD2A), anxiety (QD2A), and QoL (WHOQOL); Pretest, posttest, and 6-month F/U; Significant improvements on QD2A- depression ($p < 0.01$), QD2A- anxiety ($p < 0.05$), and physical health component of WHOQOL ($p < 0.05$) in IG compared to CG over time	Unclear

Dahlin et al. (2016); Sweden	103 adults diagnosed with generalized anxiety disorder; 39.5 years; 83.5%	- IG: 7 modules of internet-delivered ACT focusing on mindfulness, acceptance, and valued action with text, audio, animation and video + an audio CD with acceptance and mindfulness exercises and a separate workbook + support and feedback by an internet therapist for 15 min per week (*n* = 52)<br>- CG: Waitlist control (*n* = 51)	Depressive symptoms (PHQ-9), anxiety (BAI), and QoL (QOLI); Pretest, posttest, and 6-month F/U; Significant improvement on PHQ-9 and BAI in IG compared to CG over time	Low
De Wit et al. (2020); the Netherlands	148 partner caregivers of people with amyotrophic lateral sclerosis and progressive muscular atrophy; 61 years; 64.9%	- IG: A blended ACT program, involving one 1-hr face-to-face session delivered by a psychologist + 6 online guided modules of ACT with psychoeducation (1.5 hr per module) and personalized feedback by a psychologist + one 30-min telephone call with a psychologist (*n* = 74)<br>- CG: Waitlist control (*n* = 74)	Psychological distress (HADS) and QoL (CarerQoL); Pretest, posttest, and 3-month F/U; No significant between-group differences in HADS and CarerQoL over time	Low
Douma et al. (2021); the Netherlands	73 parent caregivers of children with a physical chronic illness; 42.5 years; 98.5%	- IG: 6 weekly 90-min group sessions based on ACT and CBT delivered in a secured chatroom with three to five parents guided by two psychologists + a booster session at 4 months after the last regular session, aiming to prevent and/or reduce psychosocial problems by teaching the use of adaptive disease-related coping skills (*n* = 39)<br>- CG: Waitlist control (*n* = 34)	Depressive symptoms (HADS-D), anxiety (HADS-A), and psychological distress (HADS); Baseline, 6-months F/U, and 12-months F/U; Significant improvements on HADS-D, HADS-A, and HADS total in IG compared to CG over time ($p < 0.05$)	High
Eustis et al. (2018); USA	156 college students; 25.4 years; 78.8%	- IG: A 3-session web-based therapist-assisted ACT targeting anxiety, focusing on psychoeducation, mindfulness, and values, in which each session includes two 15-min narrated PowerPoint slides with text, images, and experiential exercises and a written practice assignment about related skill practice and/or barriers to skill practice + an email reminder + written feedback and support from the therapist (*n* = 78)<br>- CG: Waitlist control (*n* = 78)	Depressive symptoms (Depression subscale of the DASS-21), anxiety (Anxiety subscale of the DASS-21), stress (Stress subscale of the DASS-21), and QoL (QOLI); Pretest, posttest, and 1-month F/U; Significant improvements on DASS-21-depression ($p<0.001$), DASS-21-stress ($p < 0.05$), and QOLI ($p < 0.05$) in IG compared to CG over time	High
Heffner et al. (2020); USA	51 daily smokers with bipolar I or II disorder; 49 years; 45%	- IG: An eight-part self-paced web-based ACT program for smoking cessation, involving ACT exercises and psychoeducation to address specific challenges to smoking cessation for smokers with bipolar disorders (WebQuit	Depressive symptoms (PHQ-9); Pretest, posttest, and 1-month F/U; No significant between-group	High

		Plus) over a 10-week treatment period + weekly email reminders containing a link to the assigned program and daily text messages for 70 days with links to the program + nicotine patch for 8 weeks (*n* = 25)<br>- CG: National Cancer Institute's Smokefree.gov, the U.S. national standard for web-based smoking cessation interventions, covering quit planning, skills training, advice on pharmacotherapy, and social support for quitting over a 10-week treatment period + weekly email reminders containing a link to the assigned program and daily text messages for 70 days with links to the program + nicotine patch for 8 weeks (*n* = 26)	differences on PHQ-9 over time	
Hesser et al. (2012); Sweden	99 adults with moderate to severe distress due to tinnitus; 48.5 years; 43.4%	- IG1: 8 weekly modules of guided internet-delivered ACT involving structured self-help material presented via the internet, an identified therapist who provided support and guidance, mindfulness and defusion exercises that could be downloaded as MP3 files, homework assignments, and email reminders (*n* = 35)<br>- IG2: 8 weekly modules of guided internet-delivered CBT in guided self-help format, including tinnitus-specific CBT techniques (e.g., applied relaxation, positive imagery,<br>attention training, cognitive restructuring), homework assignments, and email reminders (*n* = 32)<br>- CG: A moderated online discussion forum that specifically targeted tinnitus-related problems, with therapists presenting weekly discussion topics and encouraging discussion (*n* = 32)	Depressive symptoms (HADS-D), anxiety (HADS-A), stress (PSS), Tinnitus distress (THI), and QoL (QOLI); Pretest, posttest, and 12-month F/U; Significant improvements on THI ($p < 0.01$), HADS-D ($p < 0.01$), HADS-A ($p < 0.05$), and PSS ($p <0.01$) in IG1 compared to CG over time	Unclear
Hoffmann et al. (2020); Denmark	101 adults experiencing severe health anxiety (hypochondriasis); 39.8 years; 65.3%	- IG: 7 modules of a clinician-guided self-help internet-delivered ACT program over 12 weeks, developed as a web app, involving text, illustrations, audio files (mindfulness exercises), video clips, and interactive worksheets, and an encrypted and embedded message system enabling written communication (*n* = 53)<br>- CG: 7 internet-delivered discussion forums available over 12 weeks with topic related to health anxiety, such as health care, relationships, or work (*n* = 48)	Depressive symptoms (Depression subscale of the SCL-90), anxiety (WI-7), and QoL (WHO-5); Pretest, posttest, and 6-month F/U; Significant improvements on WI-7 ($p < 0.001$), depression subscale of the SCL-90 ($p < 0.001$), and WHO-5 ($p < 0.05$) in IG compared to CG over time	Low
Ivanova et al. (2016); Sweden	152 adults diagnosed with social anxiety and/or and panic	- IG1: 8 weekly modules of an internet-delivered ACT program delivered via computer and smartphone application in a therapist guided format over 10 weeks,	Depressive symptoms (PHQ-9), anxiety (GAD-7), and QoL (QOLI); Pretest,	Low

	disorder; 35.3 years; 64.5%	including short texts with voice-over, videos featuring a psychologist, video and audio exercises, images, and feedback by a therapist twice per week + a supplementary exercise book in paper format + a CD with mindfulness and acceptance exercises (*n* = 50)<br>- IG2: Same as the IG1 in unguided format (*n* = 51)<br>- CG: Waitlist control (*n* = 51)	posttest, and 12-month F/U; A significant improvement on GAD-7 in IG1 compared to CG over time ($p < 0.05$)	
Köhle et al. (2021); the Netherlands	203 partners of cancer patients; 55.9 years; 70.4%	- IG1: 6 modules (+ 2 optional modules) of web-based self-help ACT over 12 weeks, with personal feedback on participants' experiences with the module, progress in the module, and feedback on key eservices via weekly email messages from a personal counsellor (*n* = 67)<br>- IG2: 6 modules of web-based self-help ACT over 12 weeks, with short, preprogrammed feedback messages aiming to normalize and validate emotions and reactions participants could experience after completing key exercises of the module (*n* = 70)<br>- CG: Waitlist control (*n* = 66)	Stress (CSI) and psychological distress (HADS); Pretest, posttest, and 3-month F/U; No significant between-group differences on CSI and HADS over time	Low
Lappalainen et al. (2013); Finland	24 working-age males experiencing exhaustion, stress symptoms, or sleeping problems; 43.3 years; 0%	- IG: An ACT-based program that integrated different personal health technologies, including a Web portal, mobile phone applications, personal monitoring devices, and analysis software, with three 2-hr group meetings held by a psychologist (*n* = 12)<br>- CG: Waitlist control (*n* = 12)	Depressive symptoms (BDI-I), stress (BBI), and psychological distress (SCL-90); Pretest, posttest, and 6-month F/U; No significant between-group differences on BDI, BBI, and SCL-90 over time	Unclear
Lappalainen et al. (2015); Finland	39 adults with major depressive episode; 51.9 years; 71.8%	- IG: 6 weekly web-based ACT modules, involving self-help texts, videos, and downloadable MP3 audio files for mindfulness and other experiential exercises, home assignments, online personalized written feedback by trained master's-level students of psychology, and automated email-based reminders over a 7-week intervention period (*n* = 19)<br>- CG: Waitlist control (*n* = 20)	Depressive symptoms (BDI-II) and psychological distress (SCL-90); Pretest, posttest, and 12-month F/U; Significant improvements on BDI-II and SCL-90 in IG compared to CG over time ($p < 0.01$)	Unclear
Lappalainen et al. (2019); Finland	83 adults with clinical insomnia; 53.5 years; 63.9%	- IG: 6 weekly self-help web-based ACT modules for sleep disturbances with two email-based automated reminders sent every week, involving text, experiential audio exercises, and video clips (*n* = 43)<br>- CG: Waitlist control (*n* = 40)	Depressive symptoms (BDI-II) and psychological distress (SCL-90); Pretest, posttest, and 6-month F/U; A significant improvement on BDI-II in IG compared to CG over time ($p < 0.001$)	Unclear

Lappalainen et al. (2021); Finland	243 adolescents aged 15–16 years; 15.3 years; 51%	- IG1: 5 weekly modules of a web-based ACT intervention (Youth COMPASS), involving short texts, pictures, video clips, comic strips, audio-based exercises, and homework assignments, with face-to-face support (two 45-min individual face-to-face meetings) and brief weekly feedback via WhatsApp provided by a trained coach (n = 81)<br>- IG2: a 5-week web-based ACT intervention (Youth COMPASS) with brief weekly feedback via WhatsApp provided by an individual coach only (no face-to-face sessions) (n = 80)<br>- CG: Usual care (n = 82)	Depressive symptoms (DEPS); Pretest and posttest; A significant improvement on DEPS in IG1 compared to CG over time ($p < 0.05$)	Unclear
Levin et al. (2014); USA	76 undergraduate first-year students; 18.4 years; 53.9%	- IG: web-based ACT, involving two web-based lessons, such as animations, audio narration, text and graphic elements, interactive metaphors, experiential exercises, and interactive assessments, and supplementary tailored e-mails over a 3-week intervention period (n = 37)<br>- CG: Waitlist control (n = 39)	Depressive symptoms (Depression subscale of the DASS-21), anxiety (Anxiety subscale of the DASS-21), and stress (stress subscale of the DASS-21); Pretest, posttest, and 3-week F/U; No significant between-group differences on DASS-21 subscales over time	Unclear
Levin et al. (2016); USA	234 college students; 21.6 years; 76.9%	- IG: A 3-week web-based ACT program focusing on acceptance and values, involving two core multimedia sessions (audio narration, animation, text and graphic elements, and interactive exercises), supplementary e-mails, web-based resources, and text messages with reminder e-mails and phone calls (n = 114)<br>- CG: A 3-week web-based mental health education program, involving two web-based sessions that focused on providing basic educational information about the symptoms and causes of depression and anxiety and brief information on coping strategies (n = 120)	Depressive symptoms (Depression subscale of the DASS-21), anxiety (Anxiety subscale of the DASS-21), and stress (stress subscale of the DASS-21); Pretest, posttest, 1-month F/U, and 3-month F/U; No significant between-group differences on DASS-21 subscales over time	Unclear
Levin et al. (2017); USA	79 college students experiencing psychological distress; 20.5 years; 66%	- IG: 6 sessions of a web-based self-help ACT, involving text, images, audio recordings, and videos for experiential exercises, worksheets, assessments with tailored feedback, and expandable text/popup features, over a 4-week intervention period with reminder prompts via email and phone by research assistants (n = 40)<br>- CG: Waitlist control (n = 39)	Depressive symptoms (Depression subscale of CCAPS), anxiety (Anxiety subscale of the CCAPS), and psychological distress (CCAPS total); Pretest and posttest; A significant improvement on CCAPS total in IG compared to CG over time ($p < 0.05$)	Unclear

Levin et al. (2020a); USA	181 college students with clinically significant psychological distress; 22.3 years; 72.4%	- IG1: 12 self-guided web-based ACT sessions over 6 weeks, taking about 15–30 min per session, involving text, videos, audio-guided mindfulness exercises, worksheets with responsive feedback, ACT metaphors, other ACT skill practice exercises (e.g., defusion exercises), and homework assignments, with regular email prompts to support engagement and 10 min of phone coaching per week ($n = 45$)<br>- IG2: 12 self-guided web-based ACT sessions, focusing on acceptance and defusion components of ACT, over 6 weeks ($n = 45$); IG3: 12 self-guided web-based ACT sessions, focusing on values and committed action components of ACT, over 6 weeks ($n = 46$)<br>- CG: Waitlist control ($n = 45$)	Psychological distress (CCAPS); Pretest, posttest, and 1-month F/U; A significant improvement on CCAPS in IG1 compared to CG over time ($p < 0.001$)	Unclear
Levin et al. (2020b); USA	79 adults who were overweight/obese; 39.6 years; 82.3%	- IG: 8 weekly modules of an online guided self-help ACT program, integrated with nutrition education and strategies to increase physical activity, involving text, videos, and interactive exercises, with weekly 10-min phone coaching calls for support ($n = 39$)<br>- CG: Waitlist control ($n = 40$)	Psychological distress (GHQ); Pretest, posttest, and 2-month F/U; A significant improvement on GHQ in IG compared to CG over time ($p < 0.05$)	High
Lin et al. (2017); Germany	302 adults with chronic pain and pain interference; 51.7 years; 84.1%	- IG1: 8 weekly modules of internet- and mobile-based ACT with personalized and standardized feedback via e-mail within 2 working days after completion of each module by e-coaches (psychologists) ($n = 100$)<br>- IG2: 8 weekly modules of internet- and mobile-based ACT without therapist guidance ($n = 101$)<br>- CG: Waitlist control ($n = 101$)	Depressive symptoms (PHQ-9), anxiety (GAD-7), and health-related QoL (SF-12); Pretest, posttest, and 4-month F/U; No significant between-group differences on PHQ-9, GAD-7, and SF-12 over time	Low
Ly et al. (2014); Sweden	73 adults working as middle managers with staff responsibilities at a company; 41.5 years; 42.5%	- IG: 6 weekly modules of a smartphone-based ACT program for stress management, involving a short audio lecture (approximately 4–6 min), two to three texts, and two to four exercises per module, with individual feedback sent by a therapist every other day ($n = 36$)<br>- CG: Waitlist control ($n = 37$)	Stress (PSS) and psychological distress (GHQ-12); Pretest and posttest; Significant improvements on PSS and GHQ-12 in IG compared to CG over time ($p < 0.01$)	Unclear
Molander et al. (2018); Sweden	61 adults with hearing problems and psychological distress; 58.7 years; 67.2%	- IG: 8-week therapist-guided internet-based self-help ACT, involving at least weekly response on homework assignments and feedback on completed exercises using text messages in the treatment platform by a therapist ($n = 31$)<br>- CG: Waitlist control ($n = 30$)	Depressive symptoms (PHQ-9), anxiety (GAD-7), and QoL (QOLI); Pretest and posttest; Significant improvements on PHQ-9 and QOLI in IG compared to CG over time ($p < 0.05$)	Low

Muscara et al. (2020); Australia	81 parents of children with a recently diagnosed life-threatening illness or injury, reporting elevated acute stress symptoms; 37.2 years; 80.2%	- IG: A 6-session ACT-based group intervention delivered via online videoconferences over 8 weeks, involving five 90-min consecutive weekly sessions, with a sixth and final session held 3 weeks after the fifth session, delivered by two trained mental health clinicians, and provision of a session booklet and guided mindfulness CD and MP3 files (*n* = 37)<br>- CG: Waitlist control (*n* = 44)	Depressive symptoms (Depression subscale of the DASS-21), anxiety (Anxiety subscale of the DASS-21), and stress (stress subscale of the DASS-21); Pretest and posttest; No significant between-group differences on DASS-21 subscales over time	High
Pots et al. (2016); the Netherlands	236 adults with mild to moderate depressive symptoms; 46.9 years; 75.8%	- IG: 9 weekly modules of a guided web-based self-help ACT program over a 12-week intervention period, involving experiential exercises, metaphors, text messages, tailored stories, and daily 10- to 15-min mindfulness exercises provided on audio files, with personal feedback and support by a therapist via email (*n* = 82)<br>- CG1: 9 weekly modules of a guided web-based expressive writing program over a 12-week intervention period, involving writing about negative experiences, emotion regulation and reappraisal of emotions, writing about positive experiences and self-management for preventive purposes, with personal feedback and support by a therapist via email (*n* = 67)<br>- CG2: Waitlist control (*n* = 87)	Depressive symptoms (CES-D) and anxiety (HADS-A); Pretest, posttest, 3-month F/U, and 9-month F/U; Significant improvements on CES-D and HADS-A in IG compared to CG1 and CG2 over time ($p < 0.05$)	Low
Räsänen et al. (2016); Finland	68 university students experiencing psychological distress; 24.3 years; 85.3%	- IG: 7 weekly modules of a guided online ACT program, involving two face-to-face meetings with an assigned coach, online modules during a 5-week period (e.g., self-help related text, weekly wellbeing tasks, experiential exercises in audio and video format, relevant metaphors, and case study vignettes), and personal weekly written feedback and support via the website from trained student coaches (*n* = 33)<br>- CG: Waitlist control (*n* = 35)	Depressive symptoms (BDI-II), anxiety (Anxiety subscale of the DASS-21), and stress (PSS); Pretest, posttest, and 12-month F/U; Significant improvements on BDI-II and PSS in IG compared to CG over time ($p < 0.05$)	Unclear
Sagon et al. (2018); USA	103 first-year college students; 18.2 years; 71.8%	- IG: An online version of an ACT workshop offered through Blackboard throughout the semester, involving four 10- to 15-min narrated PowerPoint slides, audio recordings of guided mindfulness practices, two spaces for journal writing, and homework assignments (*n* = 52)<br>- CG: Waitlist control (*n* = 51)	Depressive symptoms (Depression subscale of the DASS-21); Pretest and posttest; No significant between-group differences on DASS-21 Depression subscale over time	Unclear
Sairanen et al. (2019); Sweden	74 parents of children with chronic conditions (type 1 diabetes: 43.2%) experiencing	- IG: 5 modules of a guided ACT web intervention over a 10-week period, involving text, videos, exercises with MP3 audio files, questionnaires, homework assignments, a discussion forum, and a free-form diary, with written feedback from	Depressive symptoms (Depression subscale of the DASS-21), anxiety (Anxiety subscale of the DASS-21), and stress (stress	Unclear

	significant burnout symptoms; 42.7 years; 81.1%	an assigned coach and an email reminder (*n* = 37)<br>- CG: Waitlist control (*n* = 37)	subscale of the DASS-21); Pretest, posttest, and 4-month F/U; A significant improvement on depression subscale of the DASS-21 in IG compared to CG over time ($p < 0.05$)	
Scott et al. (2018); UK	63 adult outpatients having chronic pain of at least 3 months duration and clinically significant pain, pain-related disability, and distress; 45.5 years; 63.5%	- IG: 8 guided online ACT sessions involving 8- to 27-min videos with individualized written feedback by a therapist and reminders over a 10- to 12-week period that started with a 30- to 45-min face-to-face or telephone session with the therapist and ended with a final face-to-face or telephone session (*n* = 31)<br>- CG: Usual care (*n* = 32)	Depressive symptoms (PHQ-9); Pretest, posttest, and 6-month F/U; A medium effect of IG on PHQ-9 compared to CG at posttest	Low
Scott et al. (2021); UK	38 adults with HIV, painful peripheral neuropathy, and at least moderate depressive symptoms; 55.9 years; 23.7%	- IG: 12 guided online ACT sessions (45–60 min each) over 8 weeks, involving brief videos and audio recordings that provided information about pain and guided participants through metaphors, experiential exercises, mindfulness practice, values clarification, and goal-setting, with individualized feedback through written messages, brief telephone calls (10–30 min), or both and phone calls for support (*n* = 25)<br>- CG: Waitlist control (*n* = 13)	Depressive symptoms (PHQ-9); Pretest, posttest, and 3-month F/U; A medium effect of IG on PHQ-9 compared to CG at posttest and F/U	Low
Simister et al. (2018); Canada	61 adults with fibromyalgia; 39.7 years; 95%	- IG: 7 modules of an online ACT program over an 8-week period, involving MP3 audio recordings, videos, metaphors, experiential exercises, introductory and recurring vignettes, and experiential homework exercises, with weekly email reminders and written feedback by the first author for clarification and positive reinforcement (*n* = 33)<br>- CG: Usual care (*n* = 34)	Depressive symptoms (CES-D); Pretest, posttest, and 3-month F/U; A significant improvement on CES-D in IG compared to CG over time ($p < 0.05$)	Low
Strandskov et al. (2017); Sweden	92 adults with bulimia nervosa or eating disorder not otherwise specified; 29.1 years; 96.7%	- IG: 8 weekly modules of a guided online ACT program, involving texts material, writing exercises, movies, audio files, homework assignments, and psychoeducation, with online feedback and support provided by a therapist (*n* = 46)<br>- CG: Waitlist control (*n* = 46)	Depressive symptoms (PHQ-9), anxiety (GAD-7), and QoL (QOLI); Pretest and posttest; No significant between-group differences on PHQ-9, GAD-7, and QOLI over time	Low
Trompetter et al. (2015); the Netherlands	238 adults with chronic pain; 54.9 years; 76%	- IG: 9 modules of an internet-delivered, guided self-help ACT program over a 9- to 12-week period, involving text, experiential exercises, metaphors, downloadable 10- to	Depressive symptoms (HADS-D) and anxiety (HADS-A); Pretest, posttest, and 3-month	Unclear

		15-min mindfulness exercises, and daily mindfulness practice, with personal feedback and support by a therapist via email (*n* = 82)<br>- CG1: 9 modules of an internet-based expressive writing program over a 9- to 12-week period (an active control), involving psychoeducation about emotions and emotion regulation related to the pain experience, writing about specific negative or positive experiences, assignments of writing at least three times a week for 15 min, and a personal diary, with personal feedback and support by a therapist via email (*n* = 79)<br>- CG2: Waitlist control (*n* = 77)	F/U; A significant improvement on HADS in IG compared to CG1 and CG2 at F/U ($p < 0.01$)	
van Aubel et al. (2020); Belgium	55 emerging adults (aged 16–25 years) with subthreshold depressive and/or psychotic complaints; 21 years; 72.7%	- IG: Blended care, involving 5 weekly face-to-face group-based ACT sessions with a trained therapist (1.5 hr per session) and daily use of the ACT-DL app between sessions for 6 weeks, providing assessment asking about current mood, context, and activities at 8 semi-random moments throughout the day for 3 subsequent days per week and options to do ACT skill practices (*n* = 27)<br>- CG: 5 weekly face-to-face group-based sessions, involving watching one of five documentaries about biography, crime, history, nature, or art and discussing several questions about the documentary (e.g., what they thought was the main message and how they could apply this message to their lives) (*n* = 28)	Depressive symptoms (IDS-SR), anxiety (STAI), and psychological distress (SCL-90); Pretest, posttest, 6-month F/U, and 12-month F/U; No significant between-group differences on IDS-SR, STAI, and SCL-90 over time	Low
Viskovich & Pakenham (2020); Australia	1162 university students; 26.9 years; 67.8%	- IG: 4 weekly modules (30–45 min per module) of a web-based ACT program, involving animated presentations, video clips, audio files, and written exercises, with nonpersonalized reminder emails to prompt program engagement and standard SMS text messages or emails to reinforce program content (*n* = 596)<br>- CG: Waitlist control (*n* = 566)	Depressive symptoms (Depression subscale of the DASS-21), anxiety (Anxiety subscale of the DASS-21), and stress (stress subscale of the DASS-21); Pretest, posttest, and 3-month F/U; Significant improvements on depression and stress subscales of DASS-21 in IG compared to CG over time ($p < 0.001$)	Unclear
Weineland et al. (2012); Sweden	39 adults who underwent bariatric surgery; 43.1 years; 89.7%	- IG: An ACT program, involving two 1.5-hr face-to-face sessions, 6 weekly internet-delivered self-help ACT modules (texts, mindfulness audio files, written exercises, and audiovisual animations), and a 30-min weekly support session via telephone (*n* = 19)<br>- CG: Usual care (*n* = 20)	QoL (WHOQOL-BREF); Pretest and posttest; A significant improvement on WHOQOL-BREF in IG compared to CG over time ($p < 0.05$)	Unclear

Witlox et al. (2021); the Netherlands	314 adults aged between 55–75 years with mild to moderately severe anxiety symptoms; 63.1 years; 61.2%	- IG: 9 modules of a web-based ACT program to be completed in 9 to 12 weeks accompanied by 4 face-to-face sessions with their mental health counselor (*n* = 150)<br>- CG: 4 face-to-face CBT sessions with daily 15- to 30-min homework assignments over 9 to 12 weeks (*n* = 164)	Depressive symptoms (PHQ-9) and anxiety (GAD-7); Pretest, posttest, 6-month F/U, and 12-month F/U; No significant between-group differences on PHQ-9 and GAD-7 over time	Unclear

Abbreviations: ACT, acceptance and commitment therapy; BAI, Beck Anxiety Inventory; BBI, Bergen Burnout Indicator; BDI, Beck Depression Inventory; CarerQoL, Care-related Quality of Life; CBT, Cognitive behavioral therapy; CCAPS, Counseling Center Assessment of Psychological Symptoms; CES-D, Center for Epidemiologic Studies Depression Scale; CG, control group; CSI, Caregiver Strain Index; DASS, Depression Anxiety Stress Scale; DEPS, Depression Scale; F/U, follow-up; GAD-7, General Anxiety Disorder-7; GHQ, General Health Questionnaire; HADS-A, Anxiety subscale of the Hospital Anxiety and Depression Scale; HADS-D, Depression subscale of the Hospital Anxiety and Depression Scale; HIV, human immunodeficiency virus; IDS-SR, Inventory of Depressive Symptomatology, Self-Report; IG, intervention group; PHQ-9, Patient Health Questionnaire-9; PSS, Perceived Stress Scale; QD2A, Questionnaire of Depression 2nd version, Abridged; QoL, quality of life; QOLI, Quality Of Life Inventory; RoB: risk of bias; SCL-90, Symptom Checklist 90; SF-12, 12-Item Short Form Health Survey; STAI, State-Trait Anxiety Inventory; THI, Tinnitus Handicap Inventory; WI-7, Whiteley Index-7; WHOQOL, World Health Organization Quality of Life Instruments; WHOQOL-BREF, Abbreviated World Health Organization Quality of Life; WHO-5, WHO-five Well-being Index.
