# Supplementary material for: Efficacy of Internet-Based Acceptance and Commitment Therapy for Depressive Symptoms, Anxiety, Stress, Psychological Distress, and Quality of Life: Systematic Review and Meta-analysis
Source: J Med Internet Res. 2022 Dec 9;24(12):e39727. doi: 10.2196/39727 (PMC9789494; doi:10.2196/39727)
Supplement: Multimedia Appendix 4 [file jmir_v24i12e39727_app4.pdf]

Study or Subgroup	iACT Mean	iACT SD	iACT Total	control Mean	control SD	control Total	Weight	Std. Mean Difference IV, Fixed, 95% CI
**4.1.1 Depressive symptoms Web-based ACT vs control at posttest**								
Buhrman et al [41], 2013	-8.85	4.4	38	-10.52	3.77	38	1.8%	0.40 [-0.05, 0.86]
Carlbring et al [30], 2013	-16.65	8.04	40	-23.43	7.67	40	1.8%	0.85 [0.40, 1.31]
Dahlin et al [34], 2016	-5.83	5.14	52	-8.33	4.63	51	2.5%	0.51 [0.11, 0.90]
Douma et al [42], 2021	-4.25	3.36	32	-5.58	4.3	31	1.5%	0.34 [-0.16, 0.84]
Eustis et al [43], 2018	-8.06	7.78	78	-12.97	10.6	78	3.7%	0.53 [0.21, 0.84]
Hesser et al [37], 2012	-3.48	2.43	33	-4.59	3.29	32	1.6%	0.38 [-0.11, 0.87]
Hoffmann et al [35], 2021	-23.3	16.51	53	-32	20.15	48	2.4%	0.47 [0.07, 0.87]
Ivanova et al [36], 2016	-4.53	4.81	42	-7.4	5	47	2.1%	0.58 [0.15, 1.00]
Lappalainen et al [31], 2015	-13.34	6.75	18	-17.85	7.34	20	0.9%	0.62 [-0.03, 1.28]
Lappalainen et al [15], 2019	-8.02	6.59	43	-11.91	9.57	40	2.0%	0.47 [0.04, 0.91]
Levin et al [16], 2014	-4.8	6.57	37	-7.04	8.42	39	1.9%	0.29 [-0.16, 0.74]
Levin et al [17], 2016	-8.79	11.59	114	-7.31	9.6	120	5.8%	-0.14 [-0.40, 0.12]
Levin et al [38], 2017	-1.51	1.16	40	-1.59	1	39	2.0%	0.07 [-0.37, 0.51]
Lin et al [46], 2017	-8.53	4.96	100	-10.38	5.21	101	4.9%	0.36 [0.08, 0.64]
Molander et al [40], 2018	-3.89	3.71	31	-5.04	2.85	30	1.5%	0.34 [-0.16, 0.85]
Pots et al [32], 2016	-14.68	8.05	82	-17.51	7.87	67	3.6%	0.35 [0.03, 0.68]
Sagon et al [18], 2018	-10	10.5	52	-13	11.52	51	2.5%	0.27 [-0.12, 0.66]
Sairanen et al [48], 2019	-8.45	8.95	37	-10.92	8.04	37	1.8%	0.29 [-0.17, 0.75]
Scott et al [33], 2021	-12.09	5.9	25	-15.89	5.34	13	0.8%	0.65 [-0.04, 1.34]
Simister et al [49], 2018	-17.76	10.83	30	-26.97	10.46	31	1.4%	0.85 [0.33, 1.38]
Strandskov et al [50], 2017	-8.25	8.18	46	-9.73	5.84	46	2.3%	0.21 [-0.20, 0.62]
Trompetter et al [51], 2015	-5.1	3.7	82	-5.7	3.8	79	4.0%	0.16 [-0.15, 0.47]
Viskovich and Pakenham [19], 2020	-9.79	5.66	596	-12.2	7.39	566	28.3%	0.37 [0.25, 0.48]
**Subtotal (95% CI)**			**1701**			**1644**	**81.1%**	**0.35 [0.28, 0.42]**
Heterogeneity: Chi² = 30.98, df = 22 (P = 0.10); I² = 29%								
Test for overall effect: Z = 9.99 (P < 0.00001)								
**4.1.2 Depressive symptoms iACT with in-person sessions vs control at posttest**								
Lappalainen et al [21], 2013	-6.18	3.31	11	-9.33	7.1	12	0.5%	0.54 [-0.30, 1.38]
Lappalainen et al [22], 2021	-5.77	6.84	64	-6.59	6.79	82	3.6%	0.12 [-0.21, 0.45]
Räsänen et al [23], 2016	-8.88	6.84	33	-12.85	5.72	35	1.6%	0.62 [0.14, 1.11]
Scott et al [24], 2018	-10.7	3.68	31	-12.4	3.88	32	1.5%	0.44 [-0.06, 0.94]
van Aubel et al [25], 2020	-19.04	1.89	27	-20.96	1.76	28	1.2%	1.04 [0.47, 1.60]
Witlox et al [27], 2021	-4.14	5.31	150	-4.76	5.16	164	7.8%	0.12 [-0.10, 0.34]
**Subtotal (95% CI)**			**316**			**353**	**16.2%**	**0.28 [0.13, 0.43]**
Heterogeneity: Chi² = 12.55, df = 5 (P = 0.03); I² = 60%								
Test for overall effect: Z = 3.59 (P = 0.0003)								
**4.1.3 Depressive symptoms Video-conferencing ACT vs control at posttest**								
Chapoutot et al [28], 2021	-2.6	3.8	15	-2.2	2.1	15	0.7%	-0.13 [-0.84, 0.59]
Muscara et al [29], 2020	-7.7	8.42	36	-10.6	7.4	44	1.9%	0.36 [-0.08, 0.81]
**Subtotal (95% CI)**			**51**			**59**	**2.7%**	**0.23 [-0.15, 0.61]**
Heterogeneity: Chi² = 1.31, df = 1 (P = 0.25); I² = 23%								
Test for overall effect: Z = 1.18 (P = 0.24)								
**Total (95% CI)**			**2068**			**2056**	**100.0%**	**0.34 [0.27, 0.40]**
Heterogeneity: Chi² = 45.79, df = 30 (P = 0.03); I² = 34%								
Test for overall effect: Z = 10.64 (P < 0.00001)								
Test for subgroup differences: Chi² = 0.95, df = 2 (P = 0.62), I² = 0%								

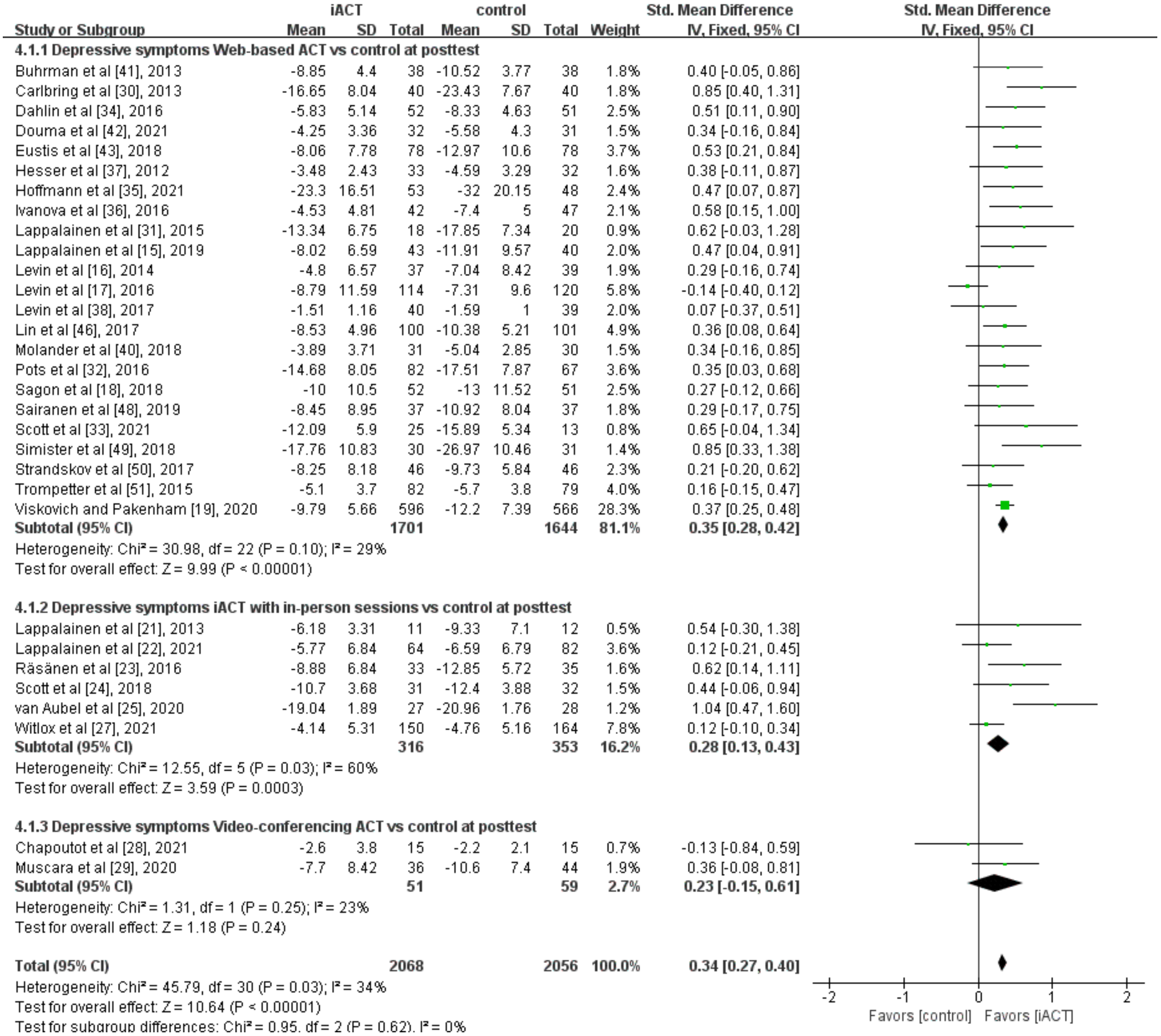

Figure S8. Forest plots showing effects of internet-based acceptance and commitment therapy on depressive symptoms according to delivery modes at the immediate posttest. iACT: internet-based acceptance and commitment therapy.

Study or Subgroup	iACT Mean	iACT SD	iACT Total	control Mean	control SD	control Total	Weight	Std. Mean Difference IV, Random, 95% CI
**4.6.1 Depressive symptoms Web-based ACT vs control at follow-up**								
Douma et al [42], 2021	-3.81	4.14	31	-4.88	4.26	32	6.5%	0.25 [-0.24, 0.75]
Eustis et al [43], 2018	-8.85	7.14	78	-12.28	10.81	78	9.0%	0.37 [0.06, 0.69]
Levin et al [16], 2014	-2.82	4.85	37	-6.15	7.23	39	7.0%	0.53 [0.07, 0.99]
Levin et al [17], 2016	-7.67	9.9	114	-5.83	7.56	120	9.9%	-0.21 [-0.47, 0.05]
Lin et al [46], 2017	-8.69	4.99	100	-10.73	5.23	101	9.6%	0.40 [0.12, 0.68]
Pots et al [32], 2016	-14.42	9.78	82	-16.06	8.78	67	8.9%	0.17 [-0.15, 0.50]
Sairanen et al [48], 2019	-6.35	9.26	37	-16.1	13.02	37	6.7%	0.85 [0.38, 1.33]
Scott et al [33], 2021	-11.36	6.15	25	-15.05	5.59	13	4.6%	0.61 [-0.08, 1.29]
Simister et al [49], 2018	-18.36	12.12	30	-25.13	12.29	31	6.3%	0.55 [0.04, 1.06]
Trompetter et al [51], 2015	-4.3	3.8	82	-6.2	3.9	79	9.1%	0.49 [0.18, 0.80]
**Subtotal (95% CI)**			**616**			**597**	**77.6%**	**0.36 [0.16, 0.57]**
Heterogeneity: Tau² = 0.06; Chi² = 25.60, df = 9 (P = 0.002); I² = 65%								
Test for overall effect: Z = 3.53 (P = 0.0004)								
**4.6.2 Depressive symptoms iACT with in-person sessions vs control at follow-up**								
Scott et al [24], 2018	-11.7	3.68	31	-12.3	3.74	32	6.5%	0.16 [-0.34, 0.65]
van Aubel et al [25], 2020	-16.8	2.3	27	-19.81	2.28	28	5.5%	1.30 [0.71, 1.88]
Witlox et al [27], 2021	-3.19	5.5	150	-4.33	5.3	164	10.4%	0.21 [-0.01, 0.43]
**Subtotal (95% CI)**			**208**			**224**	**22.4%**	**0.51 [-0.09, 1.11]**
Heterogeneity: Tau² = 0.23; Chi² = 12.01, df = 2 (P = 0.002); I² = 83%								
Test for overall effect: Z = 1.67 (P = 0.09)								
**Total (95% CI)**			**824**			**821**	**100.0%**	**0.39 [0.20, 0.57]**
Heterogeneity: Tau² = 0.07; Chi² = 37.64, df = 12 (P = 0.0002); I² = 68%								
Test for overall effect: Z = 4.12 (P < 0.0001)								
Test for subgroup differences: Chi² = 0.21, df = 1 (P = 0.64), I² = 0%								

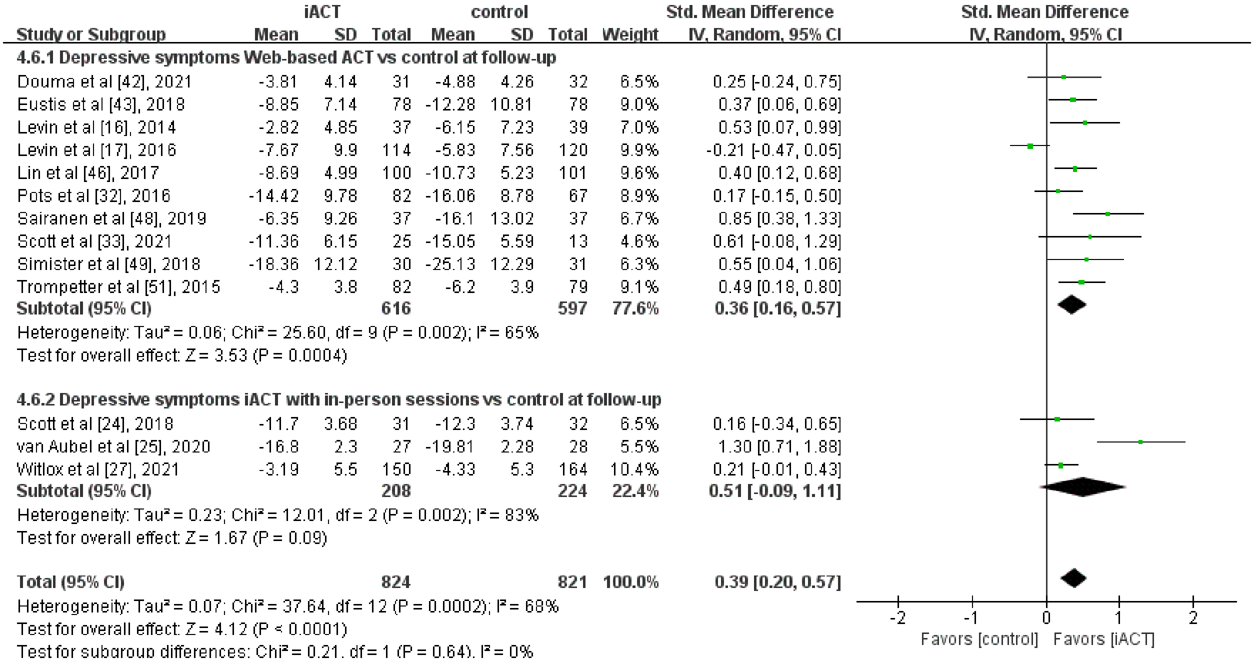

Figure S9. Forest plots showing effects of internet-based acceptance and commitment therapy on depressive symptoms according to delivery modes at follow-up. iACT: internet-based acceptance and commitment therapy.

Study or Subgroup	iACT Mean	iACT SD	iACT Total	control Mean	control SD	control Total	Weight	Std. Mean Difference IV, Random, 95% CI
**4.2.1 Anxiety Web-based ACT vs control at posttest**								
Buhrman et al [41], 2013	-8.97	4.33	38	-9.67	3.5	38	3.6%	0.18 [-0.27, 0.63]
Carlbring et al [30], 2013	-10.95	6.23	40	-13.85	6.62	40	3.6%	0.45 [0.00, 0.89]
Dahlin et al [34], 2016	-12.67	8.24	52	-17.09	7.78	51	4.2%	0.55 [0.15, 0.94]
Douma et al [42], 2021	-6.44	3.25	32	-6.81	2.97	31	3.1%	0.12 [-0.38, 0.61]
Eustis et al [43], 2018	-7.53	8.09	78	-10.51	10.8	78	5.4%	0.31 [-0.00, 0.63]
Hesser et al [37], 2012	-4.21	2.25	33	-6.78	3.98	32	3.0%	0.79 [0.28, 1.29]
Hoffmann et al [35], 2021	-31.3	20.14	53	-49	20.32	48	4.0%	0.87 [0.46, 1.28]
Ivanova et al [36], 2016	-4.81	4.17	42	-7.01	4.36	47	3.9%	0.51 [0.09, 0.93]
Levin et al [16], 2014	-2.86	3.51	37	-5.11	7.12	39	3.5%	0.39 [-0.06, 0.85]
Levin et al [17], 2016	-7.05	9.34	114	-6.94	8.68	120	6.5%	-0.01 [-0.27, 0.24]
Levin et al [38], 2017	-1.49	0.91	40	-1.63	0.97	39	3.6%	0.15 [-0.29, 0.59]
Lin et al [46], 2017	-6.11	3.91	100	-7.99	4.75	101	6.1%	0.43 [0.15, 0.71]
Molander et al [40], 2018	-3.21	3.36	31	-3.67	3.23	30	3.0%	0.14 [-0.36, 0.64]
Pots et al [32], 2016	-6.15	3.25	82	-7.47	3.23	67	5.2%	0.41 [0.08, 0.73]
Sairanen et al [48], 2019	-6.6	8.45	37	-6.04	7.14	37	3.5%	-0.07 [-0.53, 0.38]
Strandskov et al [50], 2017	-6.44	6.76	46	-7.65	4.69	46	4.0%	0.21 [-0.20, 0.62]
Trompetter et al [51], 2015	-6	3.8	82	-5.9	3.9	79	5.5%	-0.03 [-0.33, 0.28]
Viskovich and Pakenham [19], 2020	-8.4	4.94	596	-9.49	5.81	566	9.6%	0.20 [0.09, 0.32]
**Subtotal (95% CI)**			**1533**			**1489**	**81.5%**	**0.29 [0.18, 0.40]**
Heterogeneity: Tau² = 0.02; Chi² = 30.96, df = 17 (P = 0.02); I² = 45%								
Test for overall effect: Z = 5.11 (P < 0.00001)								
**4.2.2 Anxiety iACT with in-person sessions vs control at posttest**								
Räsänen et al [23], 2016	-5.79	5.42	33	-5.74	4.94	35	3.3%	-0.01 [-0.49, 0.47]
van Aubel et al [25], 2020	-44.84	2.33	27	-47.17	2.2	28	2.6%	1.01 [0.45, 1.58]
Witlox et al [27], 2021	-4.35	4.78	150	-4.76	4.61	164	7.3%	0.09 [-0.13, 0.31]
**Subtotal (95% CI)**			**210**			**227**	**13.1%**	**0.32 [-0.20, 0.84]**
Heterogeneity: Tau² = 0.17; Chi² = 9.69, df = 2 (P = 0.008); I² = 79%								
Test for overall effect: Z = 1.21 (P = 0.23)								
**4.2.3 Anxiety Video-conferencing ACT vs control at posttest**								
Chapoutot et al [28], 2021	-5	2.2	15	-5.5	1.9	15	1.7%	0.24 [-0.48, 0.96]
Muscara et al [29], 2020	-6	5.62	36	-8.4	8.72	44	3.6%	0.32 [-0.13, 0.76]
**Subtotal (95% CI)**			**51**			**59**	**5.4%**	**0.30 [-0.08, 0.67]**
Heterogeneity: Tau² = 0.00; Chi² = 0.04, df = 1 (P = 0.85); I² = 0%								
Test for overall effect: Z = 1.53 (P = 0.13)								
**Total (95% CI)**			**1794**			**1775**	**100.0%**	**0.29 [0.18, 0.39]**
Heterogeneity: Tau² = 0.03; Chi² = 41.34, df = 22 (P = 0.008); I² = 47%								
Test for overall effect: Z = 5.42 (P < 0.00001)								
Test for subgroup differences: Chi² = 0.01, df = 2 (P = 0.99), I² = 0%								

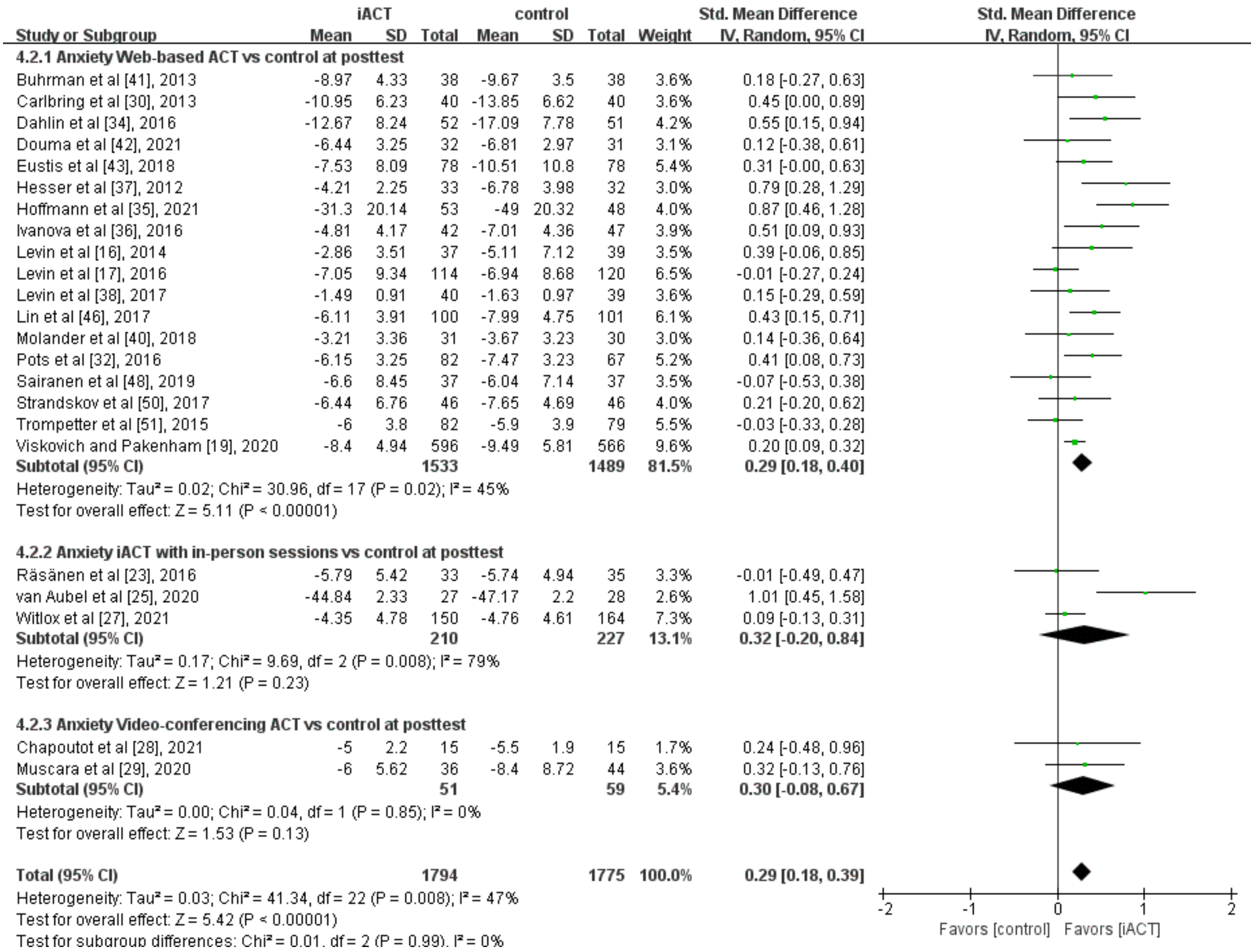

Figure S10. Forest plots showing effects of internet-based acceptance and commitment therapy on anxiety according to delivery modes at the immediate posttest. iACT: internet-based acceptance and commitment therapy.

Study or Subgroup	iACT Mean	iACT SD	iACT Total	control Mean	control SD	control Total	Weight	Std. Mean Difference IV, Fixed, 95% CI
**4.7.1 Anxiety Web-based ACT vs control at follow-up**								
Douma et al [42], 2021	-6.42	2.98	31	-6.8	3.22	32	4.3%	0.12 [-0.37, 0.62]
Eustis et al [43], 2018	-6.65	5.2	78	-11.56	10.67	78	10.2%	0.58 [0.26, 0.90]
Levin et al [16], 2014	-2.53	4.76	37	-5.19	7.06	39	5.1%	0.44 [-0.02, 0.89]
Levin et al [17], 2016	-5.4	7.39	114	-4.82	7.09	120	16.0%	-0.08 [-0.34, 0.18]
Lin et al [46], 2017	-6.29	4.05	100	-7.6	4.84	101	13.6%	0.29 [0.01, 0.57]
Pots et al [32], 2016	-6.49	3.57	82	-7.22	3.6	67	10.0%	0.20 [-0.12, 0.53]
Sairanen et al [48], 2019	-5.3	8.95	37	-11.32	15	37	4.9%	0.48 [0.02, 0.94]
Trompetter et al [51], 2015	-5.2	3.8	82	-6.2	3.8	79	10.9%	0.26 [-0.05, 0.57]
**Subtotal (95% CI)**			**561**			**553**	**75.0%**	**0.25 [0.13, 0.37]**
Heterogeneity: Chi² = 12.52, df = 7 (P = 0.08); I² = 44%								
Test for overall effect: Z = 4.11 (P < 0.0001)								
**4.7.2 Anxiety iACT with in-person sessions vs control at follow-up**								
van Aubel et al [25], 2020	-42.26	2.85	27	-43.54	2.82	28	3.7%	0.45 [-0.09, 0.98]
Witlox et al [27], 2021	-3.96	5.44	150	-4.7	5.26	164	21.4%	0.14 [-0.08, 0.36]
**Subtotal (95% CI)**			**177**			**192**	**25.0%**	**0.18 [-0.02, 0.39]**
Heterogeneity: Chi² = 1.08, df = 1 (P = 0.30); I² = 7%								
Test for overall effect: Z = 1.75 (P = 0.08)								
**Total (95% CI)**			**738**			**745**	**100.0%**	**0.23 [0.13, 0.33]**
Heterogeneity: Chi² = 13.89, df = 9 (P = 0.13); I² = 35%								
Test for overall effect: Z = 4.44 (P < 0.00001)								
Test for subgroup differences: Chi² = 0.29, df = 1 (P = 0.59), I² = 0%								

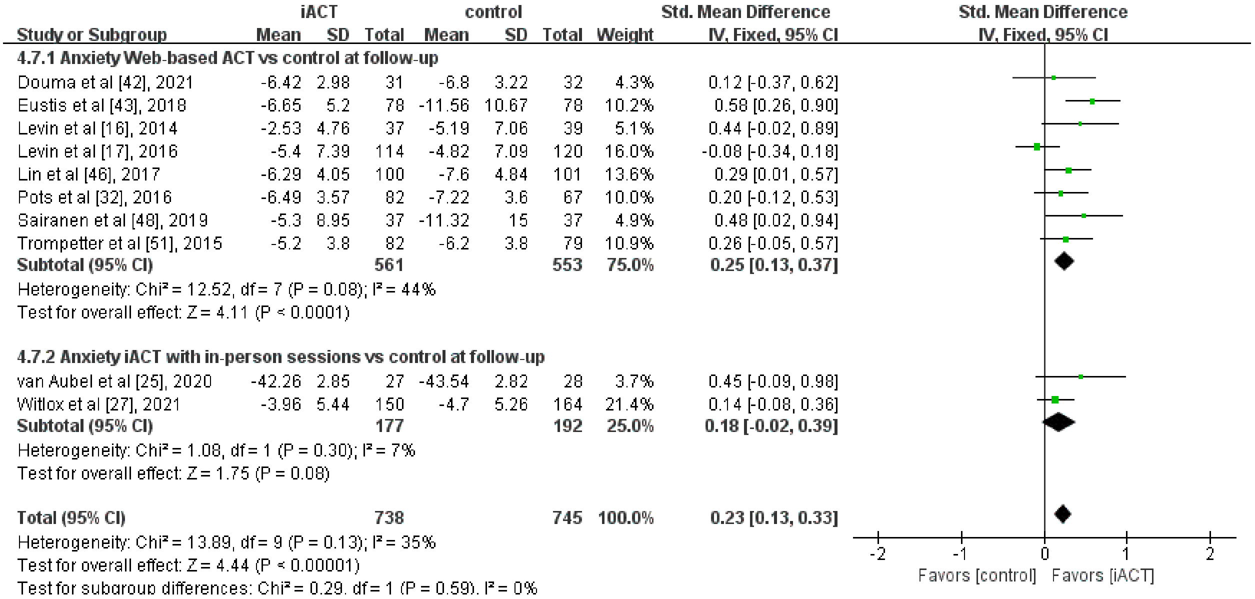

Figure S11. Forest plots showing effects of internet-based acceptance and commitment therapy on anxiety according to delivery modes at follow-up. iACT: internet-based acceptance and commitment therapy.

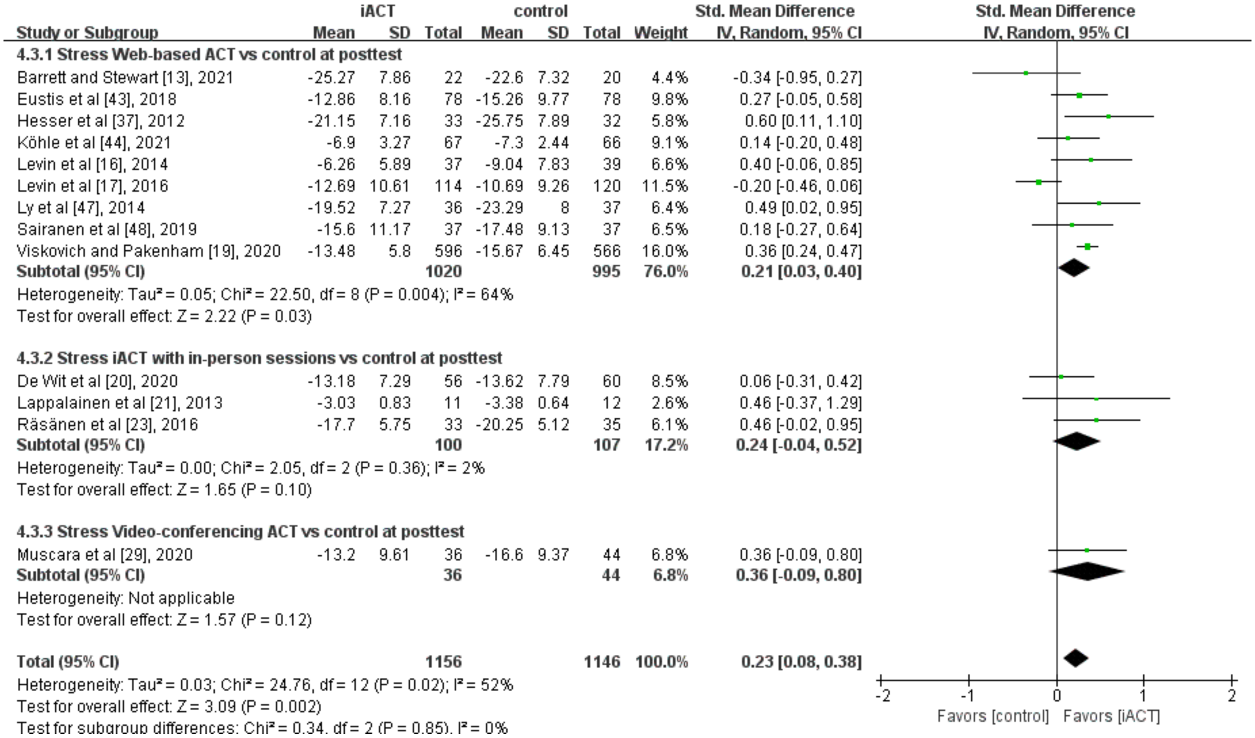

Figure S12. Forest plots showing effects of internet-based acceptance and commitment therapy on stress according to delivery modes at the immediate posttest. iACT: internet-based acceptance and commitment therapy.

Study or Subgroup	iACT Mean	iACT SD	iACT Total	control Mean	control SD	control Total	Weight	Std. Mean Difference IV, Random, 95% CI
**4.8.1 Stress Web-based ACT vs control at follow-up**								
Eustis et al [43], 2018	-12.71	5.92	78	-16.07	11.52	78	21.9%	0.37 [0.05, 0.68]
Levin et al [16], 2014	-4.53	6.06	37	-8.7	6.52	39	17.4%	0.66 [0.19, 1.12]
Levin et al [17], 2016	-9.88	9.61	114	-9.02	8.35	120	23.7%	-0.10 [-0.35, 0.16]
Sairanen et al [48], 2019	-12.25	10.57	37	-20.32	12.41	37	17.2%	0.69 [0.22, 1.16]
**Subtotal (95% CI)**			**266**			**274**	**80.2%**	**0.37 [-0.02, 0.76]**
Heterogeneity: Tau² = 0.12; Chi² = 13.83, df = 3 (P = 0.003); I² = 78%								
Test for overall effect: Z = 1.87 (P = 0.06)								
**4.8.2 Stress iACT with in-person sessions vs control at follow-up**								
De Wit et al [20], 2020	-13.89	7.76	46	-14.2	7.58	60	19.8%	0.04 [-0.34, 0.42]
**Subtotal (95% CI)**			**46**			**60**	**19.8%**	**0.04 [-0.34, 0.42]**
Heterogeneity: Not applicable								
Test for overall effect: Z = 0.21 (P = 0.84)								
**Total (95% CI)**			**312**			**334**	**100.0%**	**0.30 [-0.01, 0.61]**
Heterogeneity: Tau² = 0.09; Chi² = 14.73, df = 4 (P = 0.005); I² = 73%								
Test for overall effect: Z = 1.87 (P = 0.06)								
Test for subgroup differences: Chi² = 1.41, df = 1 (P = 0.24), I² = 28.9%								

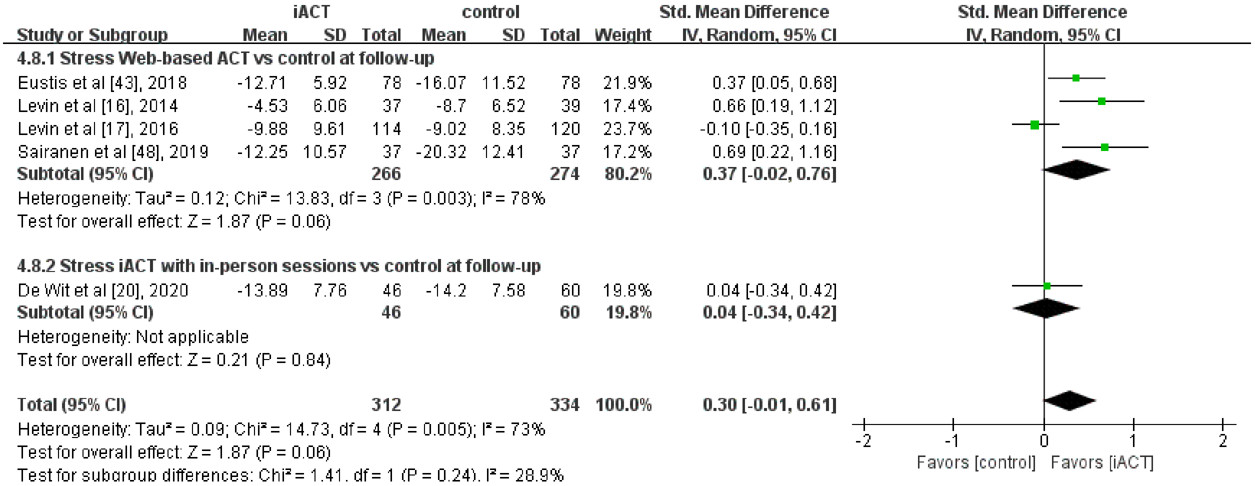

Figure S13. Forest plots showing effects of internet-based acceptance and commitment therapy on stress according to delivery modes at follow-up. iACT: internet-based acceptance and commitment therapy.

Study or Subgroup	iACT Mean	iACT SD	iACT Total	control Mean	control SD	control Total	Weight	Std. Mean Difference IV, Random, 95% CI
**4.4.1 Psychological distress Web-based ACT vs control at posttest**								
Barrett and Stewart [13], 2021	-12.41	7.07	22	-10.5	6.54	20	6.6%	-0.27 [-0.88, 0.33]
Douma et al [42], 2021	-10.69	5.94	32	-12.39	6.53	31	7.8%	0.27 [-0.23, 0.77]
Hesser et al [37], 2012	-31.94	14.54	33	-49.94	16.09	32	7.4%	1.16 [0.63, 1.69]
Köhle et al [44], 2021	-12.4	6.55	67	-14.7	6.5	66	9.4%	0.35 [0.01, 0.69]
Lappalainen et al [31], 2015	-0.65	0.44	18	-0.98	0.54	20	6.2%	0.65 [-0.00, 1.31]
Lappalainen et al [15], 2019	-0.41	0.36	43	-0.59	0.54	40	8.4%	0.39 [-0.04, 0.83]
Levin et al [38], 2017	-1.52	0.91	40	-1.6	0.78	39	8.3%	0.09 [-0.35, 0.53]
Levin et al [39], 2020	-1.26	0.67	45	-1.54	0.74	45	8.6%	0.39 [-0.02, 0.81]
Levin et al [45], 2020	-22.63	5.87	34	-26.03	5.39	38	8.0%	0.60 [0.12, 1.07]
Ly et al [47], 2014	-8.03	4.5	36	-10.09	5.71	37	8.1%	0.40 [-0.07, 0.86]
**Subtotal (95% CI)**			**370**			**368**	**78.9%**	**0.40 [0.20, 0.60]**
Heterogeneity: Tau² = 0.05; Chi² = 16.13, df = 9 (P = 0.06); I² = 44%								
Test for overall effect: Z = 3.92 (P < 0.0001)								
**4.4.2 Psychological distress iACT with in-person sessions vs control at posttest**								
De Wit et al [20], 2020	-10.75	7.62	56	-11.37	7.7	60	9.2%	0.08 [-0.28, 0.44]
Lappalainen et al [21], 2013	-0.4	0.18	11	-0.51	0.36	12	4.8%	0.37 [-0.46, 1.19]
van Aubel et al [25], 2020	-166.6	8.83	27	-158.54	8.35	28	7.1%	-0.93 [-1.48, -0.37]
**Subtotal (95% CI)**			**94**			**100**	**21.1%**	**-0.18 [-0.92, 0.56]**
Heterogeneity: Tau² = 0.34; Chi² = 10.45, df = 2 (P = 0.005); I² = 81%								
Test for overall effect: Z = 0.48 (P = 0.63)								
**Total (95% CI)**			**464**			**468**	**100.0%**	**0.28 [0.04, 0.51]**
Heterogeneity: Tau² = 0.12; Chi² = 37.57, df = 12 (P = 0.0002); I² = 68%								
Test for overall effect: Z = 2.28 (P = 0.02)								
Test for subgroup differences: Chi² = 2.22, df = 1 (P = 0.14), I² = 55.0%								

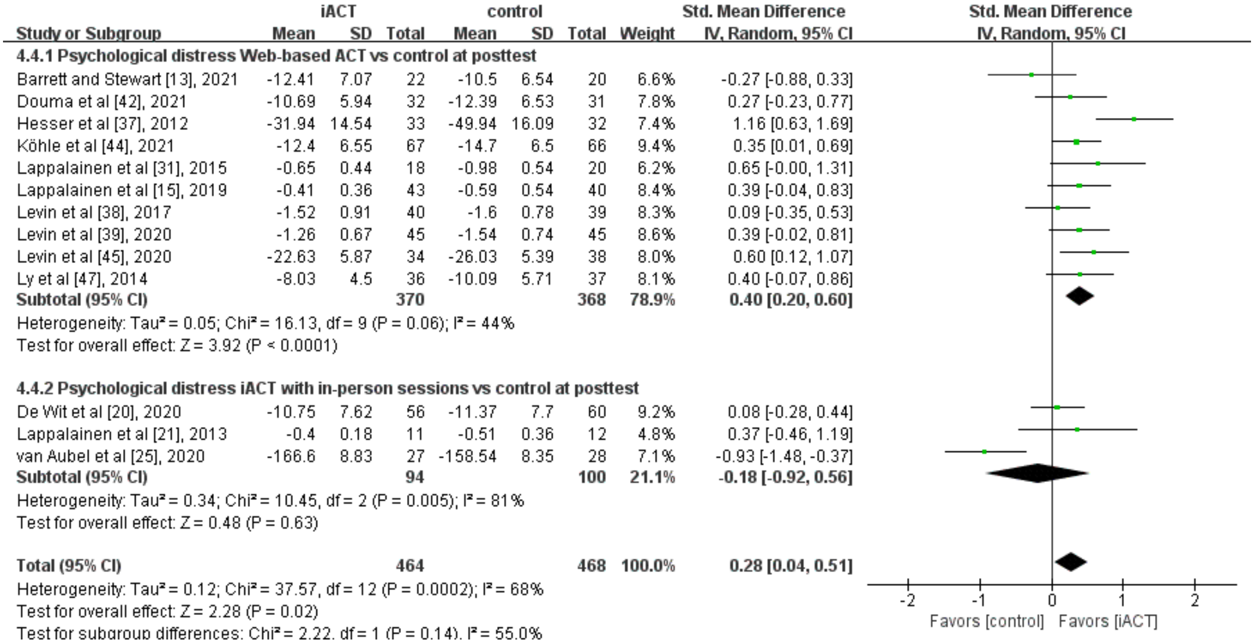

Figure S14. Forest plots showing effects of internet-based acceptance and commitment therapy on psychological distress according to delivery modes at the immediate posttest. iACT: internet-based acceptance and commitment therapy.

	iACT			control				Std. Mean Difference
Study or Subgroup	Mean	SD	Total	Mean	SD	Total	Weight	IV, Fixed, 95% CI
**4.9.1 Psychological distress Web-based ACT vs control at follow-up**								
Douma et al [42], 2021	-10.23	6.75	31	-11.68	6.75	32	20.4%	0.21 [-0.28, 0.71]
Levin et al [39], 2020	-1.13	0.8	45	-1.57	0.8	45	28.3%	0.55 [0.12, 0.97]
**Subtotal (95% CI)**			**76**			**77**	**48.7%**	**0.41 [0.08, 0.73]**
Heterogeneity: Chi² = 1.01, df = 1 (P = 0.32); I² = 1%								
Test for overall effect: Z = 2.48 (P = 0.01)								
**4.9.2 Psychological distress iACT with in-person sessions vs control at follow-up**								
De Wit et al [20], 2020	-11	7.71	46	-11.48	7.42	60	34.0%	0.06 [-0.32, 0.45]
van Aubel et al [25], 2020	-151.12	11.85	27	-157.48	11.31	28	17.3%	0.54 [0.00, 1.08]
**Subtotal (95% CI)**			**73**			**88**	**51.3%**	**0.22 [-0.09, 0.54]**
Heterogeneity: Chi² = 2.01, df = 1 (P = 0.16); I² = 50%								
Test for overall effect: Z = 1.41 (P = 0.16)								
**Total (95% CI)**			**149**			**165**	**100.0%**	**0.31 [0.09, 0.54]**
Heterogeneity: Chi² = 3.64, df = 3 (P = 0.30); I² = 18%								
Test for overall effect: Z = 2.74 (P = 0.006)								
Test for subgroup differences: Chi² = 0.63, df = 1 (P = 0.43), I² = 0%								

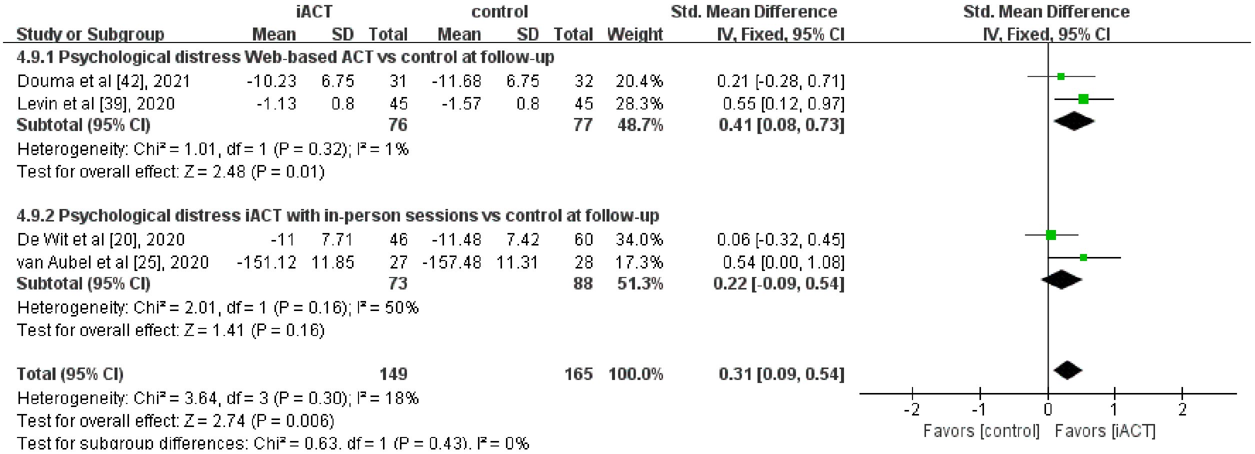

Figure S15. Forest plots showing effects of internet-based acceptance and commitment therapy on psychological distress according to delivery modes at follow-up. iACT: internet-based acceptance and commitment therapy.

Study or Subgroup	iACT Mean	iACT SD	iACT Total	control Mean	control SD	control Total	Weight	Std. Mean Difference IV, Fixed, 95% CI
**4.5.1 Quality of life Web-based ACT vs control at posttest**								
Buhrman et al [41], 2013	0.56	2.07	38	0.39	1.77	38	6.3%	0.09 [-0.36, 0.54]
Carlbring et al [30], 2013	0.78	1.57	40	0.75	1.77	40	6.6%	0.02 [-0.42, 0.46]
Dahlin et al [34], 2016	1.68	1.33	52	1.51	1.4	51	8.5%	0.12 [-0.26, 0.51]
Eustis et al [43], 2018	2.35	1.6	78	1.46	1.69	78	12.4%	0.54 [0.22, 0.86]
Hesser et al [37], 2012	2.12	1.47	33	2.27	1.5	32	5.4%	-0.10 [-0.59, 0.39]
Hoffmann et al [35], 2021	55.6	18.14	53	43.8	18.42	48	7.9%	0.64 [0.24, 1.04]
Ivanova et al [36], 2016	1.21	1.7	42	0.84	1.57	47	7.3%	0.22 [-0.19, 0.64]
Lin et al [46], 2017	48.46	10.12	100	43.85	11.07	101	16.2%	0.43 [0.15, 0.71]
Molander et al [40], 2018	2.65	1.57	31	1.87	1.71	30	4.9%	0.47 [-0.04, 0.98]
Strandskov et al [50], 2017	1.11	2.11	46	1.36	1.42	46	7.6%	-0.14 [-0.55, 0.27]
**Subtotal (95% CI)**			**513**			**511**	**83.1%**	**0.28 [0.15, 0.40]**
Heterogeneity: Chi² = 16.42, df = 9 (P = 0.06); I² = 45%								
Test for overall effect: Z = 4.36 (P < 0.0001)								
**4.5.2 Quality of life iACT with in-person sessions vs control at posttest**								
De Wit et al [20], 2020	10.63	2.23	56	10.1	2.28	60	9.5%	0.23 [-0.13, 0.60]
Lappalainen et al [21], 2013	74.91	11.64	11	69.92	14.49	12	1.9%	0.36 [-0.46, 1.19]
Weineland et al [26], 2012	94.53	11.95	19	87.05	12.26	20	3.1%	0.61 [-0.04, 1.25]
**Subtotal (95% CI)**			**86**			**92**	**14.4%**	**0.33 [0.03, 0.63]**
Heterogeneity: Chi² = 0.98, df = 2 (P = 0.61); I² = 0%								
Test for overall effect: Z = 2.17 (P = 0.03)								
**4.5.3 Quality of life Video-conferencing ACT vs control at posttest**								
Chapoutot et al [28], 2021	65.9	13.3	15	60	14.8	15	2.4%	0.41 [-0.32, 1.13]
**Subtotal (95% CI)**			**15**			**15**	**2.4%**	**0.41 [-0.32, 1.13]**
Heterogeneity: Not applicable								
Test for overall effect: Z = 1.10 (P = 0.27)								
**Total (95% CI)**			**614**			**618**	**100.0%**	**0.29 [0.17, 0.40]**
Heterogeneity: Chi² = 17.61, df = 13 (P = 0.17); I² = 26%								
Test for overall effect: Z = 4.97 (P < 0.00001)								
Test for subgroup differences: Chi² = 0.22, df = 2 (P = 0.90), I² = 0%								

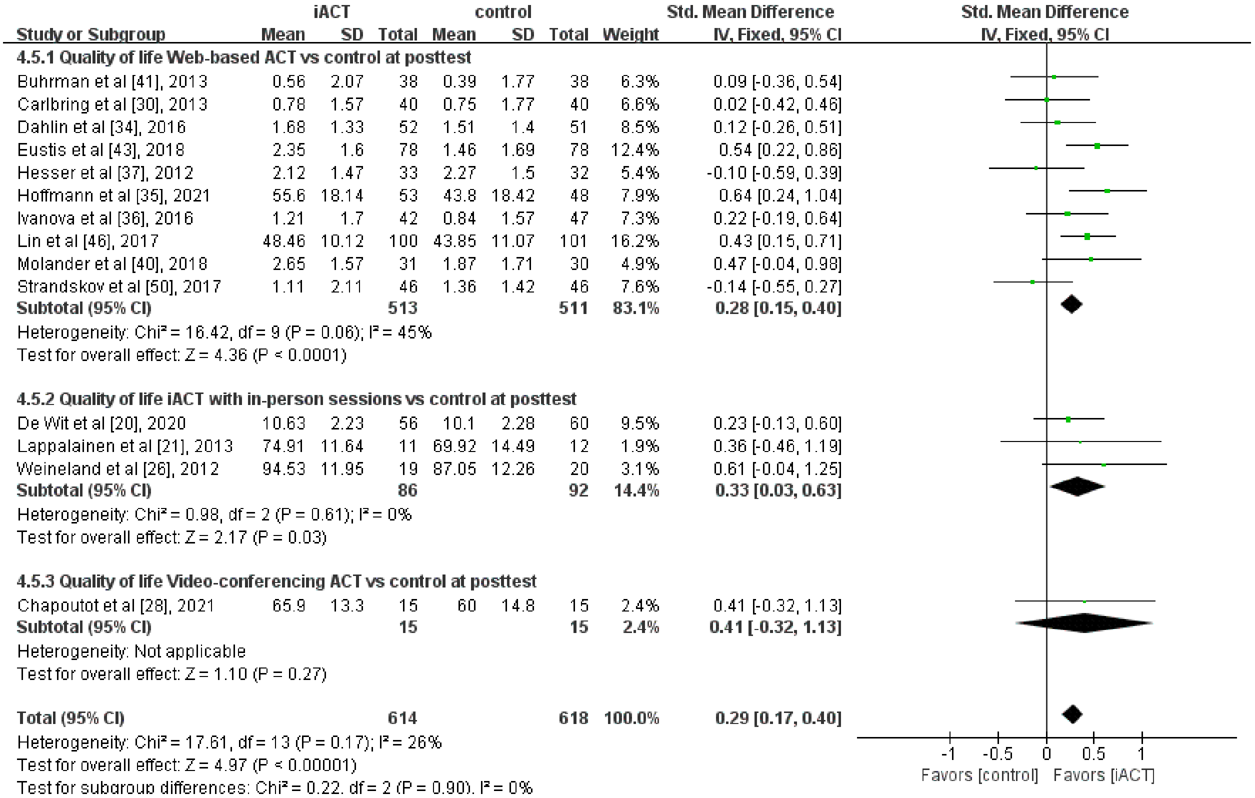

Figure S16. Forest plots showing effects of internet-based acceptance and commitment therapy on quality of life according to delivery modes at the immediate posttest. iACT: internet-based acceptance and commitment therapy.

Study or Subgroup	iACT Mean	iACT SD	iACT Total	control Mean	control SD	control Total	Weight	Std. Mean Difference IV, Fixed, 95% CI
**4.10.1 Quality of life Web-based ACT vs control at follow-up**								
Eustis et al [43], 2018	1.61	1.68	78	1.41	2.01	78	34.1%	0.11 [-0.21, 0.42]
Lin et al [46], 2017	47.35	9.27	100	43.45	10.83	101	43.2%	0.39 [0.11, 0.66]
**Subtotal (95% CI)**			**178**			**179**	**77.2%**	**0.26 [0.05, 0.47]**
Heterogeneity: Chi² = 1.68, df = 1 (P = 0.19); I² = 40%								
Test for overall effect: Z = 2.47 (P = 0.01)								
**4.10.2 Quality of life iACT with in-person sessions vs control at follow-up**								
De Wit et al [20], 2020	10.09	2.23	46	9.97	2.09	60	22.8%	0.06 [-0.33, 0.44]
**Subtotal (95% CI)**			**46**			**60**	**22.8%**	**0.06 [-0.33, 0.44]**
Heterogeneity: Not applicable								
Test for overall effect: Z = 0.28 (P = 0.78)								
**Total (95% CI)**			**224**			**239**	**100.0%**	**0.22 [0.03, 0.40]**
Heterogeneity: Chi² = 2.54, df = 2 (P = 0.28); I² = 21%								
Test for overall effect: Z = 2.30 (P = 0.02)								
Test for subgroup differences: Chi² = 0.86, df = 1 (P = 0.35), I² = 0%								

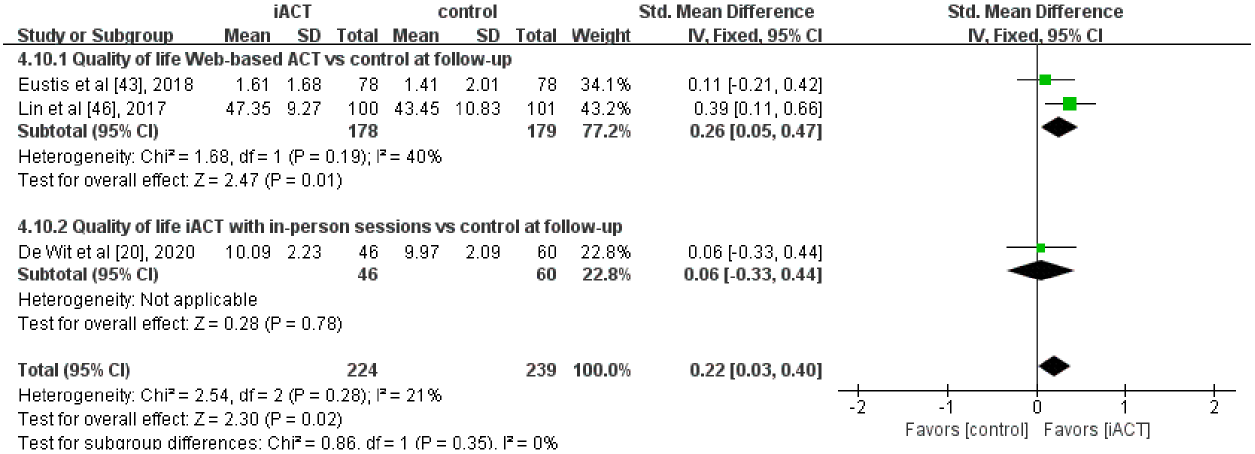

Figure S17. Forest plots showing effects of internet-based acceptance and commitment therapy on quality of life according to delivery modes at follow-up. iACT: internet-based acceptance and commitment therapy.
