# Supplementary material for: Efficacy of Internet-Based Acceptance and Commitment Therapy for Depressive Symptoms, Anxiety, Stress, Psychological Distress, and Quality of Life: Systematic Review and Meta-analysis
Source: J Med Internet Res. 2022 Dec 9;24(12):e39727. doi: 10.2196/39727 (PMC9789494; doi:10.2196/39727)
Supplement: Multimedia Appendix 5 [file jmir_v24i12e39727_app5.pdf]

Study or Subgroup	iACT Mean	iACT SD	iACT Total	control Mean	control SD	control Total	Weight	Std. Mean Difference IV, Fixed, 95% CI
**5.1.1 Depressive symptoms iACT that were targeted for people with depressive symptoms vs control at posttest**								
Carlbring et al [30], 2013	-16.65	8.04	40	-23.43	7.67	40	1.8%	0.85 [0.40, 1.31]
Lappalainen et al [31], 2015	-13.34	6.75	18	-17.85	7.34	20	0.9%	0.62 [-0.03, 1.28]
Pots et al [32], 2016	-14.68	8.05	82	-17.51	7.87	67	3.6%	0.35 [0.03, 0.68]
Scott et al [33], 2021	-12.09	5.9	25	-15.89	5.34	13	0.8%	0.65 [-0.04, 1.34]
van Aubel et al [25], 2020	-19.04	1.89	27	-20.96	1.76	28	1.2%	1.04 [0.47, 1.60]
**Subtotal (95% CI)**			**192**			**168**	**8.3%**	**0.62 [0.40, 0.83]**
Heterogeneity: Chi² = 5.68, df = 4 (P = 0.22); I² = 30%								
Test for overall effect: Z = 5.66 (P < 0.00001)								
**5.1.2 Depressive symptoms iACT that were not targeted for people with depressive symptoms vs control at posttest**								
Buhrman et al [41], 2013	-8.85	4.4	38	-10.52	3.77	38	1.8%	0.40 [-0.05, 0.86]
Chapoutot et al [28], 2021	-2.6	3.8	15	-2.2	2.1	15	0.7%	-0.13 [-0.84, 0.59]
Dahlin et al [34], 2016	-5.83	5.14	52	-8.33	4.63	51	2.5%	0.51 [0.11, 0.90]
Douma et al [42], 2021	-4.25	3.36	32	-5.58	4.3	31	1.5%	0.34 [-0.16, 0.84]
Eustis et al [43], 2018	-8.06	7.78	78	-12.97	10.6	78	3.7%	0.53 [0.21, 0.84]
Hesser et al [37], 2012	-3.48	2.43	33	-4.59	3.29	32	1.6%	0.38 [-0.11, 0.87]
Hoffmann et al [35], 2021	-23.3	16.51	53	-32	20.15	48	2.4%	0.47 [0.07, 0.87]
Ivanova et al [36], 2016	-4.53	4.81	42	-7.4	5	47	2.1%	0.58 [0.15, 1.00]
Lappalainen et al [21], 2013	-6.18	3.31	11	-9.33	7.1	12	0.5%	0.54 [-0.30, 1.38]
Lappalainen et al [15], 2019	-8.02	6.59	43	-11.91	9.57	40	2.0%	0.47 [0.04, 0.91]
Lappalainen et al [22], 2021	-5.77	6.84	64	-6.59	6.79	82	3.6%	0.12 [-0.21, 0.45]
Levin et al [16], 2014	-4.8	6.57	37	-7.04	8.42	39	1.9%	0.29 [-0.16, 0.74]
Levin et al [17], 2016	-8.79	11.59	114	-7.31	9.6	120	5.8%	-0.14 [-0.40, 0.12]
Levin et al [38], 2017	-1.51	1.16	40	-1.59	1	39	2.0%	0.07 [-0.37, 0.51]
Lin et al [46], 2017	-8.53	4.96	100	-10.38	5.21	101	4.9%	0.36 [0.08, 0.64]
Molander et al [40], 2018	-3.89	3.71	31	-5.04	2.85	30	1.5%	0.34 [-0.16, 0.85]
Muscara et al [29], 2020	-7.7	8.42	36	-10.6	7.4	44	1.9%	0.36 [-0.08, 0.81]
Räsänen et al [23], 2016	-8.88	6.84	33	-12.85	5.72	35	1.6%	0.62 [0.14, 1.11]
Sagon et al [18], 2018	-10	10.5	52	-13	11.52	51	2.5%	0.27 [-0.12, 0.66]
Sairanen et al [48], 2019	-8.45	8.95	37	-10.92	8.04	37	1.8%	0.29 [-0.17, 0.75]
Scott et al [24], 2018	-10.7	3.68	31	-12.4	3.88	32	1.5%	0.44 [-0.06, 0.94]
Simister et al [49], 2018	-17.76	10.83	30	-26.97	10.46	31	1.4%	0.85 [0.33, 1.38]
Strandskov et al [50], 2017	-8.25	8.18	46	-9.73	5.84	46	2.3%	0.21 [-0.20, 0.62]
Trompetter et al [51], 2015	-5.1	3.7	82	-5.7	3.8	79	4.0%	0.16 [-0.15, 0.47]
Viskovich and Pakenham [19], 2020	-9.79	5.66	596	-12.2	7.39	566	28.3%	0.37 [0.25, 0.48]
Witlox et al [27], 2021	-4.14	5.31	150	-4.76	5.16	164	7.8%	0.12 [-0.10, 0.34]
**Subtotal (95% CI)**			**1876**			**1888**	**91.7%**	**0.31 [0.25, 0.37]**
Heterogeneity: Chi² = 32.77, df = 25 (P = 0.14); I² = 24%								
Test for overall effect: Z = 9.41 (P < 0.00001)								
**Total (95% CI)**			**2068**			**2056**	**100.0%**	**0.34 [0.27, 0.40]**
Heterogeneity: Chi² = 45.79, df = 30 (P = 0.03); I² = 34%								
Test for overall effect: Z = 10.64 (P < 0.00001)								
Test for subgroup differences: Chi² = 7.34, df = 1 (P = 0.007), I² = 86.4%								

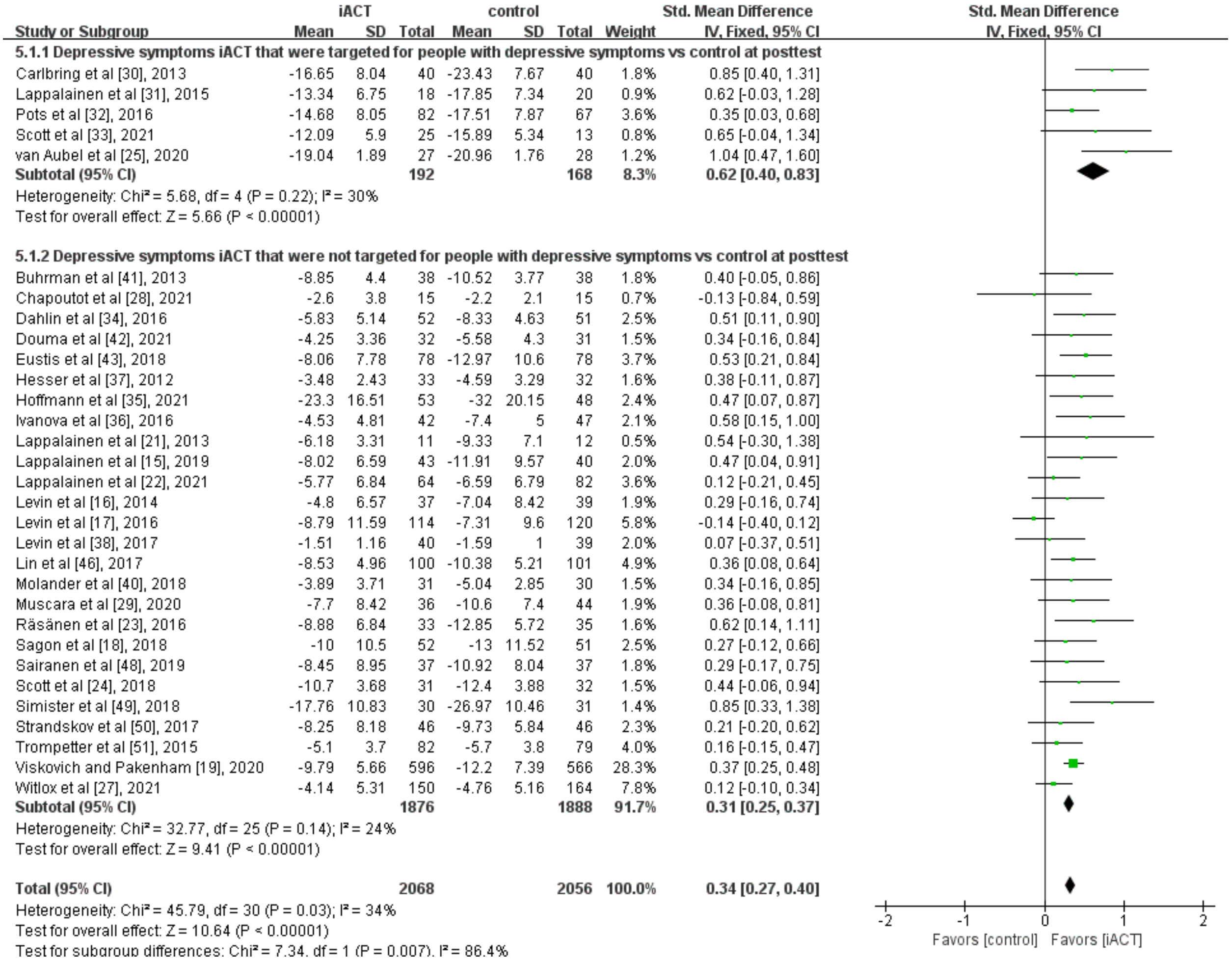

Figure S18. Forest plots showing effects of internet-based acceptance and commitment therapy on depressive symptoms according to the use of targeted participants at the immediate posttest. iACT: internet-based acceptance and commitment therapy.

Study or Subgroup	iACT Mean	iACT SD	iACT Total	control Mean	control SD	control Total	Weight	Std. Mean Difference IV, Random, 95% CI
**5.5.1 Depressive symptoms iACT that were targeted for people with depressive symptoms vs control at follow-up**								
Pots et al [32], 2016	-14.42	9.78	82	-16.06	8.78	67	8.9%	0.17 [-0.15, 0.50]
Scott et al [33], 2021	-11.36	6.15	25	-15.05	5.59	13	4.6%	0.61 [-0.08, 1.29]
van Aubel et al [25], 2020	-16.8	2.3	27	-19.81	2.28	28	5.5%	1.30 [0.71, 1.88]
**Subtotal (95% CI)**			**134**			**108**	**19.0%**	**0.66 [-0.04, 1.37]**
Heterogeneity: Tau² = 0.31; Chi² = 11.00, df = 2 (P = 0.004); I² = 82%								
Test for overall effect: Z = 1.84 (P = 0.07)								
**5.5.2 Depressive symptoms iACT that were not targeted for people with depressive symptoms vs control at follow-up**								
Douma et al [42], 2021	-3.81	4.14	31	-4.88	4.26	32	6.5%	0.25 [-0.24, 0.75]
Eustis et al [43], 2018	-8.85	7.14	78	-12.28	10.81	78	9.0%	0.37 [0.06, 0.69]
Levin et al [16], 2014	-2.82	4.85	37	-6.15	7.23	39	7.0%	0.53 [0.07, 0.99]
Levin et al [17], 2016	-7.67	9.9	114	-5.83	7.56	120	9.9%	-0.21 [-0.47, 0.05]
Lin et al [46], 2017	-8.69	4.99	100	-10.73	5.23	101	9.6%	0.40 [0.12, 0.68]
Sairanen et al [48], 2019	-6.35	9.26	37	-16.1	13.02	37	6.7%	0.85 [0.38, 1.33]
Scott et al [24], 2018	-11.7	3.68	31	-12.3	3.74	32	6.5%	0.16 [-0.34, 0.65]
Simister et al [49], 2018	-18.36	12.12	30	-25.13	12.29	31	6.3%	0.55 [0.04, 1.06]
Trompetter et al [51], 2015	-4.3	3.8	82	-6.2	3.9	79	9.1%	0.49 [0.18, 0.80]
Witlox et al [27], 2021	-3.19	5.5	150	-4.33	5.3	164	10.4%	0.21 [-0.01, 0.43]
**Subtotal (95% CI)**			**690**			**713**	**81.0%**	**0.33 [0.15, 0.52]**
Heterogeneity: Tau² = 0.05; Chi² = 25.04, df = 9 (P = 0.003); I² = 64%								
Test for overall effect: Z = 3.50 (P = 0.0005)								
**Total (95% CI)**			**824**			**821**	**100.0%**	**0.39 [0.20, 0.57]**
Heterogeneity: Tau² = 0.07; Chi² = 37.64, df = 12 (P = 0.0002); I² = 68%								
Test for overall effect: Z = 4.12 (P < 0.0001)								
Test for subgroup differences: Chi² = 0.79, df = 1 (P = 0.37), I² = 0%								

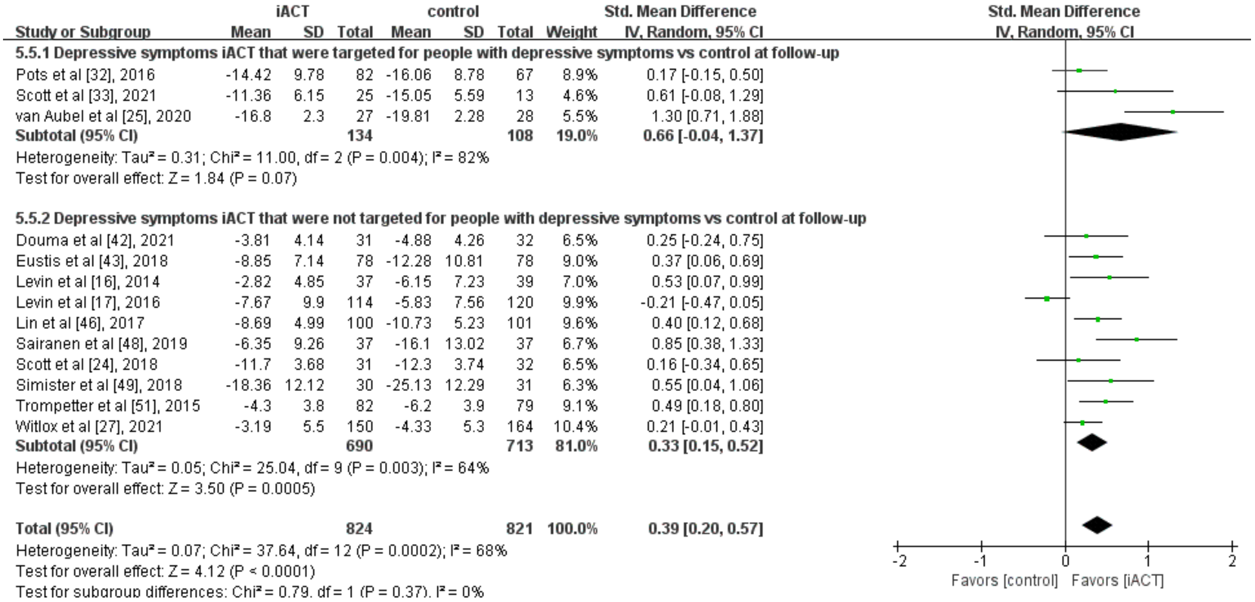

Figure S19. Forest plots showing effects of internet-based acceptance and commitment therapy on depressive symptoms according to the use of targeted participants at follow-up. iACT: internet-based acceptance and commitment therapy.

Study or Subgroup	iACT Mean	iACT SD	iACT Total	control Mean	control SD	control Total	Weight	Std. Mean Difference IV, Random, 95% CI
**5.2.1 Anxiety iACT that were targeted for people with anxiety vs control at posttest**								
Dahlin et al [34], 2016	-12.67	8.24	52	-17.09	7.78	51	4.2%	0.55 [0.15, 0.94]
Hoffmann et al [35], 2021	-31.3	20.14	53	-49	20.32	48	4.0%	0.87 [0.46, 1.28]
Ivanova et al [36], 2016	-4.81	4.17	42	-7.01	4.36	47	3.9%	0.51 [0.09, 0.93]
Witlox et al [27], 2021	-4.35	4.78	150	-4.76	4.61	164	7.3%	0.09 [-0.13, 0.31]
**Subtotal (95% CI)**			**297**			**310**	**19.4%**	**0.48 [0.11, 0.85]**
Heterogeneity: Tau² = 0.11; Chi² = 13.09, df = 3 (P = 0.004); I² = 77%								
Test for overall effect: Z = 2.55 (P = 0.01)								
**5.2.2 Anxiety iACT that were not targeted for people with anxiety vs control at posttest**								
Buhrman et al [41], 2013	-8.97	4.33	38	-9.67	3.5	38	3.6%	0.18 [-0.27, 0.63]
Carlbring et al [30], 2013	-10.95	6.23	40	-13.85	6.62	40	3.6%	0.45 [0.00, 0.89]
Chapoutot et al [28], 2021	-5	2.2	15	-5.5	1.9	15	1.7%	0.24 [-0.48, 0.96]
Douma et al [42], 2021	-6.44	3.25	32	-6.81	2.97	31	3.1%	0.12 [-0.38, 0.61]
Eustis et al [43], 2018	-7.53	8.09	78	-10.51	10.8	78	5.4%	0.31 [-0.00, 0.63]
Hesser et al [37], 2012	-4.21	2.25	33	-6.78	3.98	32	3.0%	0.79 [0.28, 1.29]
Levin et al [16], 2014	-2.86	3.51	37	-5.11	7.12	39	3.5%	0.39 [-0.06, 0.85]
Levin et al [17], 2016	-7.05	9.34	114	-6.94	8.68	120	6.5%	-0.01 [-0.27, 0.24]
Levin et al [38], 2017	-1.49	0.91	40	-1.63	0.97	39	3.6%	0.15 [-0.29, 0.59]
Lin et al [46], 2017	-6.11	3.91	100	-7.99	4.75	101	6.1%	0.43 [0.15, 0.71]
Molander et al [40], 2018	-3.21	3.36	31	-3.67	3.23	30	3.0%	0.14 [-0.36, 0.64]
Muscara et al [29], 2020	-6	5.62	36	-8.4	8.72	44	3.6%	0.32 [-0.13, 0.76]
Pots et al [32], 2016	-6.15	3.25	82	-7.47	3.23	67	5.2%	0.41 [0.08, 0.73]
Räsänen et al [23], 2016	-5.79	5.42	33	-5.74	4.94	35	3.3%	-0.01 [-0.49, 0.47]
Sairanen et al [48], 2019	-6.6	8.45	37	-6.04	7.14	37	3.5%	-0.07 [-0.53, 0.38]
Strandskov et al [50], 2017	-6.44	6.76	46	-7.65	4.69	46	4.0%	0.21 [-0.20, 0.62]
Trompetter et al [51], 2015	-6	3.8	82	-5.9	3.9	79	5.5%	-0.03 [-0.33, 0.28]
van Aubel et al [25], 2020	-44.84	2.33	27	-47.17	2.2	28	2.6%	1.01 [0.45, 1.58]
Viskovich and Pakenham [19], 2020	-8.4	4.94	596	-9.49	5.81	566	9.6%	0.20 [0.09, 0.32]
**Subtotal (95% CI)**			**1497**			**1465**	**80.6%**	**0.24 [0.14, 0.35]**
Heterogeneity: Tau² = 0.01; Chi² = 26.46, df = 18 (P = 0.09); I² = 32%								
Test for overall effect: Z = 4.70 (P < 0.00001)								
**Total (95% CI)**			**1794**			**1775**	**100.0%**	**0.29 [0.18, 0.39]**
Heterogeneity: Tau² = 0.03; Chi² = 41.34, df = 22 (P = 0.008); I² = 47%								
Test for overall effect: Z = 5.42 (P < 0.00001)								
Test for subgroup differences: Chi² = 1.44, df = 1 (P = 0.23), I² = 30.5%								

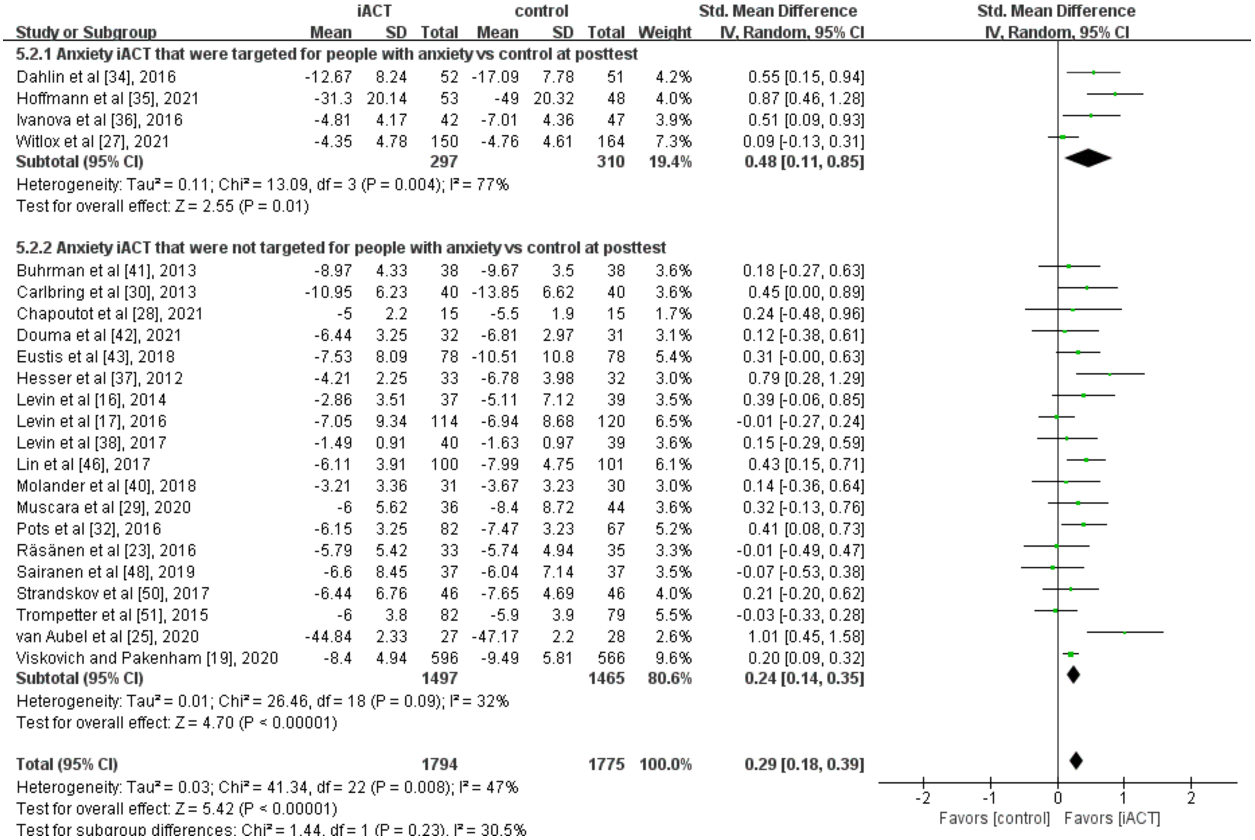

Figure S20. Forest plots showing effects of internet-based acceptance and commitment therapy on anxiety according to the use of targeted participants at the immediate posttest. iACT: internet-based acceptance and commitment therapy.

Study or Subgroup	iACT Mean	iACT SD	iACT Total	control Mean	control SD	control Total	Weight	Std. Mean Difference IV, Fixed, 95% CI
**5.6.1 Anxiety iACT that were targeted for people with anxiety vs control at follow-up**								
Witlox et al [27], 2021	-3.96	5.44	150	-4.7	5.26	164	21.4%	0.14 [-0.08, 0.36]
**Subtotal (95% CI)**			**150**			**164**	**21.4%**	**0.14 [-0.08, 0.36]**
Heterogeneity: Not applicable								
Test for overall effect: Z = 1.22 (P = 0.22)								
**5.6.2 Anxiety iACT that were not targeted for people with anxiety vs control at follow-up**								
Douma et al [42], 2021	-6.42	2.98	31	-6.8	3.22	32	4.3%	0.12 [-0.37, 0.62]
Eustis et al [43], 2018	-6.65	5.2	78	-11.56	10.67	78	10.2%	0.58 [0.26, 0.90]
Levin et al [16], 2014	-2.53	4.76	37	-5.19	7.06	39	5.1%	0.44 [-0.02, 0.89]
Levin et al [17], 2016	-5.4	7.39	114	-4.82	7.09	120	16.0%	-0.08 [-0.34, 0.18]
Lin et al [46], 2017	-6.29	4.05	100	-7.6	4.84	101	13.6%	0.29 [0.01, 0.57]
Pots et al [32], 2016	-6.49	3.57	82	-7.22	3.6	67	10.0%	0.20 [-0.12, 0.53]
Sairanen et al [48], 2019	-5.3	8.95	37	-11.32	15	37	4.9%	0.48 [0.02, 0.94]
Trompetter et al [51], 2015	-5.2	3.8	82	-6.2	3.8	79	10.9%	0.26 [-0.05, 0.57]
van Aubel et al [25], 2020	-42.26	2.85	27	-43.54	2.82	28	3.7%	0.45 [-0.09, 0.98]
**Subtotal (95% CI)**			**588**			**581**	**78.6%**	**0.26 [0.14, 0.37]**
Heterogeneity: Chi² = 13.02, df = 8 (P = 0.11); I² = 39%								
Test for overall effect: Z = 4.37 (P < 0.0001)								
**Total (95% CI)**			**738**			**745**	**100.0%**	**0.23 [0.13, 0.33]**
Heterogeneity: Chi² = 13.89, df = 9 (P = 0.13); I² = 35%								
Test for overall effect: Z = 4.44 (P < 0.00001)								
Test for subgroup differences: Chi² = 0.88, df = 1 (P = 0.35), I² = 0%								

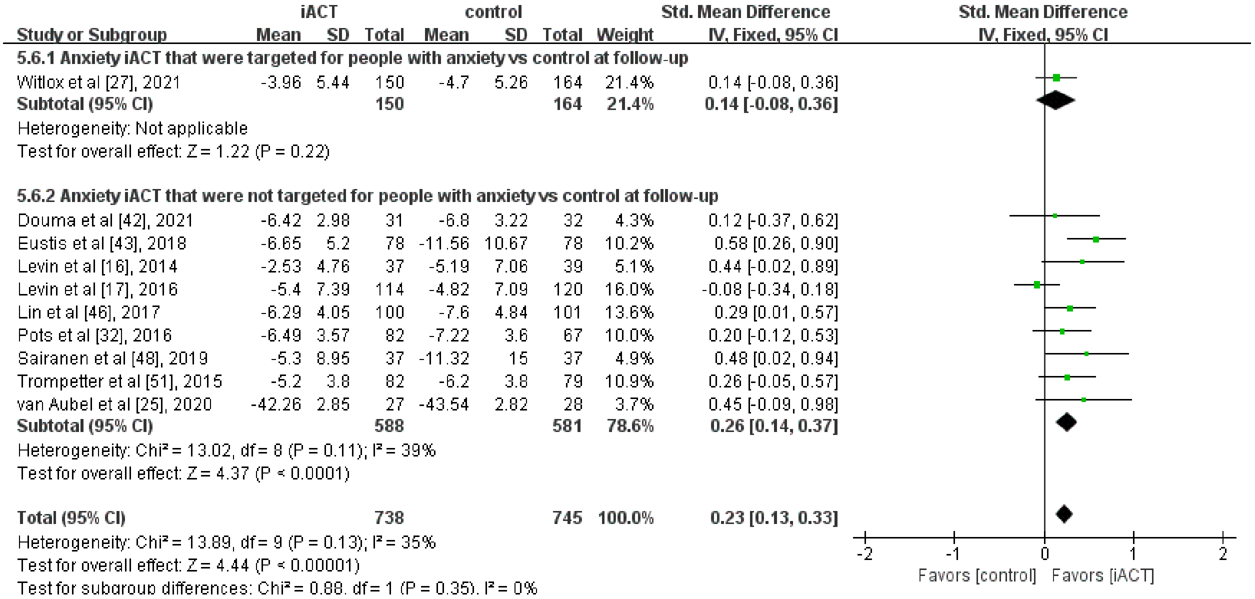

Figure S21. Forest plots showing effects of internet-based acceptance and commitment therapy on anxiety according to the use of targeted participants at follow-up. iACT: internet-based acceptance and commitment therapy.

Study or Subgroup	iACT Mean	iACT SD	iACT Total	control Mean	control SD	control Total	Weight	Std. Mean Difference IV, Fixed, 95% CI
**5.3.1 Stress iACT that were targeted for people with stress vs control at posttest**								
Lappalainen et al [21], 2013	-3.03	0.83	11	-3.38	0.64	12	1.0%	0.46 [-0.37, 1.29]
Muscara et al [29], 2020	-13.2	9.61	36	-16.6	9.37	44	3.4%	0.36 [-0.09, 0.80]
**Subtotal (95% CI)**			**47**			**56**	**4.4%**	**0.38 [-0.01, 0.77]**
Heterogeneity: Chi² = 0.05, df = 1 (P = 0.83); I² = 0%								
Test for overall effect: Z = 1.89 (P = 0.06)								
**5.3.2 Stress iACT that were not targeted for people with stress vs control at posttest**								
Barrett and Stewart [13], 2021	-25.27	7.86	22	-22.6	7.32	20	1.8%	-0.34 [-0.95, 0.27]
De Wit et al [20], 2020	-13.18	7.29	56	-13.62	7.79	60	5.1%	0.06 [-0.31, 0.42]
Eustis et al [43], 2018	-12.86	8.16	78	-15.26	9.77	78	6.8%	0.27 [-0.05, 0.58]
Hesser et al [37], 2012	-21.15	7.16	33	-25.75	7.89	32	2.7%	0.60 [0.11, 1.10]
Köhle et al [44], 2021	-6.9	3.27	67	-7.3	2.44	66	5.9%	0.14 [-0.20, 0.48]
Levin et al [16], 2014	-6.26	5.89	37	-9.04	7.83	39	3.3%	0.40 [-0.06, 0.85]
Levin et al [17], 2016	-12.69	10.61	114	-10.69	9.26	120	10.3%	-0.20 [-0.46, 0.06]
Ly et al [47], 2014	-19.52	7.27	36	-23.29	8	37	3.1%	0.49 [0.02, 0.95]
Räsänen et al [23], 2016	-17.7	5.75	33	-20.25	5.12	35	2.9%	0.46 [-0.02, 0.95]
Sairanen et al [48], 2019	-15.6	11.17	37	-17.48	9.13	37	3.2%	0.18 [-0.27, 0.64]
Viskovich and Pakenham [19], 2020	-13.48	5.8	596	-15.67	6.45	566	50.4%	0.36 [0.24, 0.47]
**Subtotal (95% CI)**			**1109**			**1090**	**95.6%**	**0.26 [0.17, 0.34]**
Heterogeneity: Chi² = 24.37, df = 10 (P = 0.007); I² = 59%								
Test for overall effect: Z = 6.00 (P < 0.00001)								
**Total (95% CI)**			**1156**			**1146**	**100.0%**	**0.26 [0.18, 0.35]**
Heterogeneity: Chi² = 24.76, df = 12 (P = 0.02); I² = 52%								
Test for overall effect: Z = 6.27 (P < 0.00001)								
Test for subgroup differences: Chi² = 0.35, df = 1 (P = 0.56), I² = 0%								

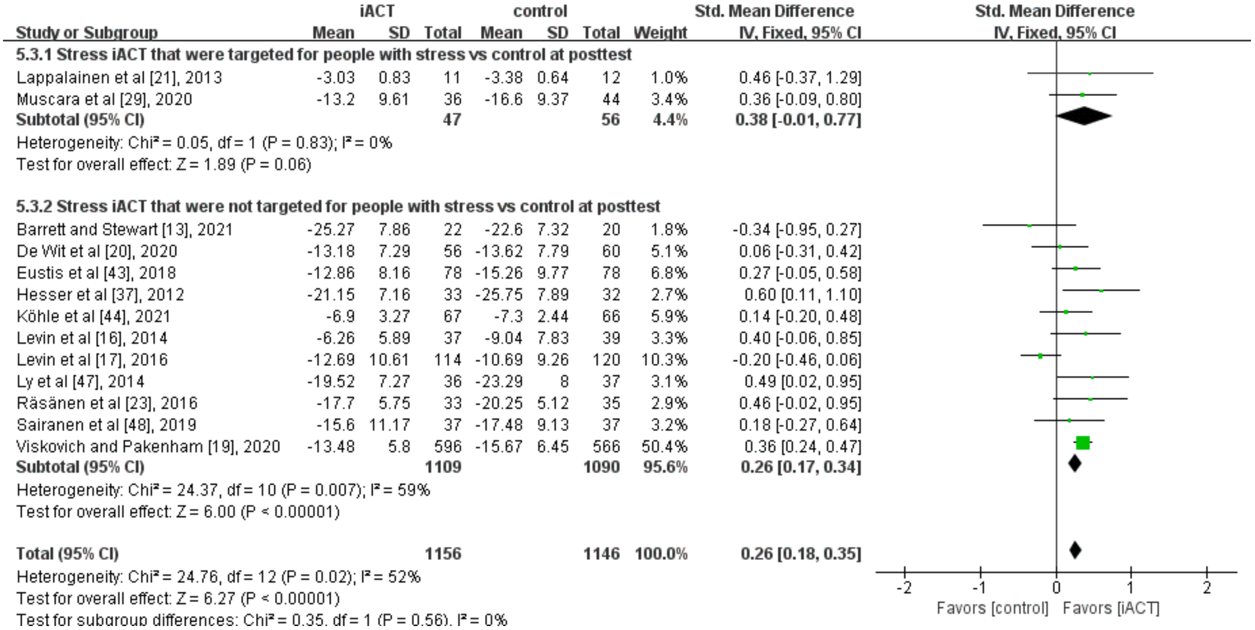

Figure S22. Forest plots showing effects of internet-based acceptance and commitment therapy on stress according to the use of targeted participants at the immediate posttest. iACT: internet-based acceptance and commitment therapy.

Study or Subgroup	iACT Mean	iACT SD	iACT Total	control Mean	control SD	control Total	Weight	Std. Mean Difference IV, Random, 95% CI
**5.4.1 Psychological distress iACT that were targeted for people with psychological distress vs control at posttest**								
Hesser et al [37], 2012	-31.94	14.54	33	-49.94	16.09	32	7.4%	1.16 [0.63, 1.69]
Levin et al [38], 2017	-1.52	0.91	40	-1.6	0.78	39	8.3%	0.09 [-0.35, 0.53]
Levin et al [39], 2020	-1.26	0.67	45	-1.54	0.74	45	8.6%	0.39 [-0.02, 0.81]
**Subtotal (95% CI)**			**118**			**116**	**24.4%**	**0.53 [-0.05, 1.11]**
Heterogeneity: Tau² = 0.21; Chi² = 9.50, df = 2 (P = 0.009); I² = 79%								
Test for overall effect: Z = 1.80 (P = 0.07)								
**5.4.2 Psychological distress iACT that were not targeted for people with psychological distress vs control at posttest**								
Barrett and Stewart [13], 2021	-12.41	7.07	22	-10.5	6.54	20	6.6%	-0.27 [-0.88, 0.33]
De Wit et al [20], 2020	-10.75	7.62	56	-11.37	7.7	60	9.2%	0.08 [-0.28, 0.44]
Douma et al [42], 2021	-10.69	5.94	32	-12.39	6.53	31	7.8%	0.27 [-0.23, 0.77]
Köhle et al [44], 2021	-12.4	6.55	67	-14.7	6.5	66	9.4%	0.35 [0.01, 0.69]
Lappalainen et al [21], 2013	-0.4	0.18	11	-0.51	0.36	12	4.8%	0.37 [-0.46, 1.19]
Lappalainen et al [31], 2015	-0.65	0.44	18	-0.98	0.54	20	6.2%	0.65 [-0.00, 1.31]
Lappalainen et al [15], 2019	-0.41	0.36	43	-0.59	0.54	40	8.4%	0.39 [-0.04, 0.83]
Levin et al [45], 2020	-22.63	5.87	34	-26.03	5.39	38	8.0%	0.60 [0.12, 1.07]
Ly et al [47], 2014	-8.03	4.5	36	-10.09	5.71	37	8.1%	0.40 [-0.07, 0.86]
van Aubel et al [25], 2020	-166.6	8.83	27	-158.54	8.35	28	7.1%	-0.93 [-1.48, -0.37]
**Subtotal (95% CI)**			**346**			**352**	**75.6%**	**0.20 [-0.06, 0.46]**
Heterogeneity: Tau² = 0.11; Chi² = 25.25, df = 9 (P = 0.003); I² = 64%								
Test for overall effect: Z = 1.48 (P = 0.14)								
**Total (95% CI)**			**464**			**468**	**100.0%**	**0.28 [0.04, 0.51]**
Heterogeneity: Tau² = 0.12; Chi² = 37.57, df = 12 (P = 0.0002); I² = 68%								
Test for overall effect: Z = 2.28 (P = 0.02)								
Test for subgroup differences: Chi² = 1.07, df = 1 (P = 0.30), I² = 6.3%								

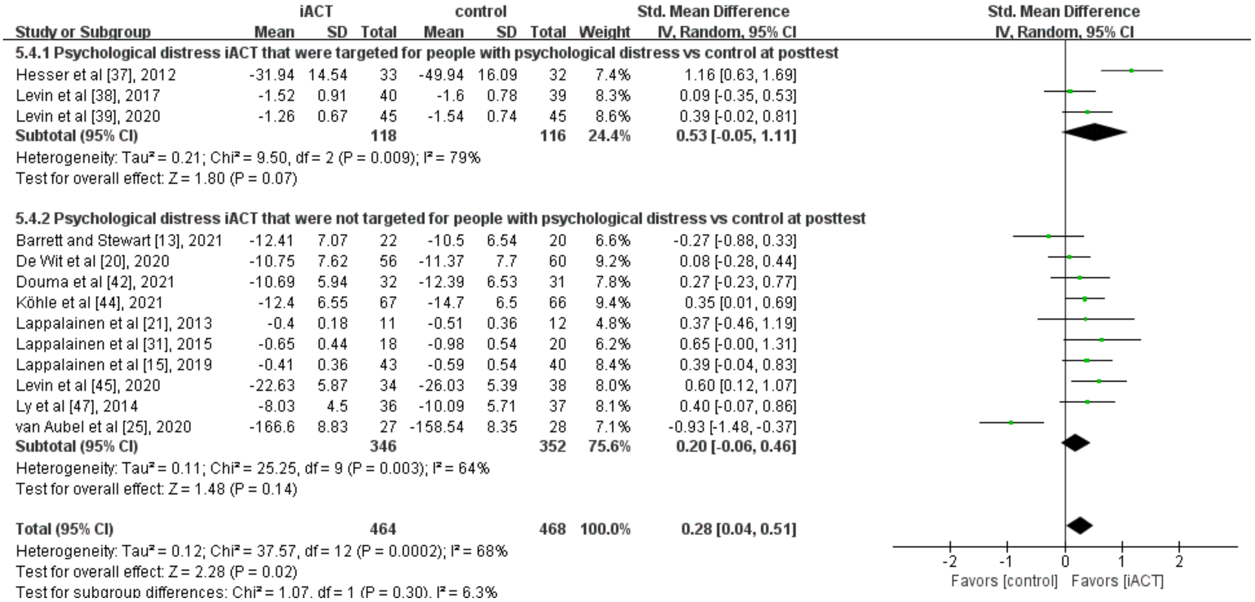

Figure S23. Forest plots showing effects of internet-based acceptance and commitment therapy on psychological distress according to the use of targeted participants at the immediate posttest. iACT: internet-based acceptance and commitment therapy.

Study or Subgroup	iACT Mean	iACT SD	iACT Total	control Mean	control SD	control Total	Weight	Std. Mean Difference IV, Fixed, 95% CI
**5.7.1 Psychological distress iACT that were targeted for people with psychological distress vs control at follow-up**								
Levin et al [39], 2020	-1.13	0.8	45	-1.57	0.8	45	28.3%	0.55 [0.12, 0.97]
**Subtotal (95% CI)**			**45**			**45**	**28.3%**	**0.55 [0.12, 0.97]**
Heterogeneity: Not applicable								
Test for overall effect: Z = 2.54 (P = 0.01)								
**5.7.2 Psychological distress iACT that were not targeted for people with psychological distress vs control at follow-up**								
De Wit et al [20], 2020	-11	7.71	46	-11.48	7.42	60	34.0%	0.06 [-0.32, 0.45]
Douma et al [42], 2021	-10.23	6.75	31	-11.68	6.75	32	20.4%	0.21 [-0.28, 0.71]
van Aubel et al [25], 2020	-151.12	11.85	27	-157.48	11.31	28	17.3%	0.54 [0.00, 1.08]
**Subtotal (95% CI)**			**104**			**120**	**71.7%**	**0.22 [-0.04, 0.49]**
Heterogeneity: Chi² = 2.01, df = 2 (P = 0.37); I² = 0%								
Test for overall effect: Z = 1.64 (P = 0.10)								
**Total (95% CI)**			**149**			**165**	**100.0%**	**0.31 [0.09, 0.54]**
Heterogeneity: Chi² = 3.64, df = 3 (P = 0.30); I² = 18%								
Test for overall effect: Z = 2.74 (P = 0.006)								
Test for subgroup differences: Chi² = 1.63, df = 1 (P = 0.20), I² = 38.8%								

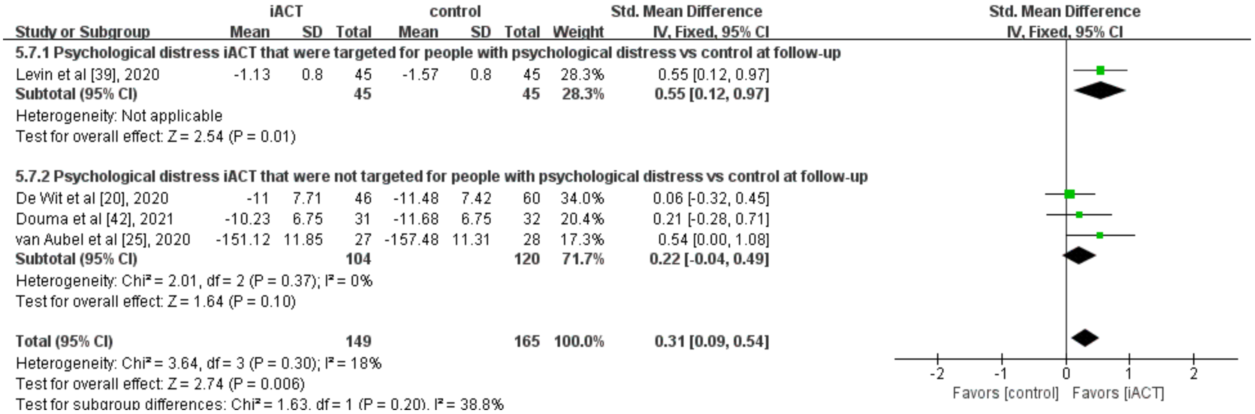

Figure S24. Forest plots showing effects of internet-based acceptance and commitment therapy on psychological distress according to the use of targeted participants at follow-up. iACT: internet-based acceptance and commitment therapy.
