# Supplementary material for: Efficacy of Internet-Based Acceptance and Commitment Therapy for Depressive Symptoms, Anxiety, Stress, Psychological Distress, and Quality of Life: Systematic Review and Meta-analysis
Source: J Med Internet Res. 2022 Dec 9;24(12):e39727. doi: 10.2196/39727 (PMC9789494; doi:10.2196/39727)
Supplement: Multimedia Appendix 6 [file jmir_v24i12e39727_app6.pdf]

Risk of Bias (RoB) of the Included Studies

Reference	Random Sequence Generation	Allocation Concealment	Blinding of Participants and Personnel	Blinding of Outcome Assessment	Incomplete Outcome Data	Selective Reporting	Overall RoB
Barrett & Stewart (2021)	Low	Unclear	Unclear	Low	Low	Unclear	Unclear
Buhrman et al. (2013)	Low	Low	High	Low	Low	Low	Low
Carlbring et al. (2013)	Low	Low	Unclear	Low	Low	Unclear	Unclear
Chapoutot et al. (2021)	Low	Unclear	Unclear	Low	Low	Unclear	Unclear
Dahlin et al. (2016)	Low	Low	Unclear	Low	Low	Low	Low
De Wit et al. (2020)	Low	Low	High	Low	Low	Low	Low
Douma et al. (2021)	Low	Low	High	Low	High	Low	High
Eustis et al. (2018)	Low	High	Unclear	Low	Low	Unclear	High
Heffner et al. (2020)	Unclear	Unclear	High	Low	Low	High	High
Hesser et al. (2012)	Low	Low	Unclear	Low	Low	Unclear	Unclear
Hoffmann et al. (2020)	Low	Low	Unclear	Low	Low	Low	Low
Ivanova et al. (2016)	Low	Low	Unclear	Low	Low	Low	Low
Köhle et al. (2021)	Low	Low	High	Low	Low	Low	Low
Lappalainen et al. (2013)	Low	Unclear	High	Low	Low	Unclear	Unclear
Lappalainen et al. (2015)	Low	Low	Unclear	Low	Low	Unclear	Unclear
Lappalainen et al. (2019)	Low	Low	Unclear	Low	Low	Unclear	Unclear
Lappalainen et al. (2021)	Low	Low	Unclear	Low	Low	Unclear	Unclear
Levin et al. (2014)	Low	Unclear	Unclear	Low	Low	Unclear	Unclear
Levin et al. (2016)	Low	Low	Low	Low	Low	Unclear	Unclear
Levin et al. (2017)	Low	Low	High	Low	Low	Unclear	Unclear
Levin et al. (2020a)	Low	Low	Unclear	Low	Low	Unclear	Unclear
Levin et al. (2020b)	Unclear	Unclear	Unclear	Low	High	Low	High
Lin et al. (2017)	Low	Low	Unclear	Low	Low	Low	Low
Ly et al. (2014)	Low	Low	Unclear	Low	Low	Unclear	Unclear
Molander et al. (2018)	Low	Low	Unclear	Low	Low	Low	Low
Muscara et al. (2020)	Low	Low	Low	Low	High	Low	High
Pots et al. (2016)	Low	Low	Unclear	Low	Low	Low	Low

Räsänen et al. (2016)	Low	Low	Unclear	Low	Low	Unclear	Unclear
Sagon et al. (2018)	Unclear	Unclear	Unclear	Low	Low	Unclear	Unclear
Sairanen et al. (2019)	Low	Low	Unclear	Low	Low	Unclear	Unclear
Scott et al. (2018)	Low	Low	High	Low	Low	Low	Low
Scott et al. (2021)	Low	Low	High	Low	Low	Low	Low
Simister et al. (2018)	Low	Low	Unclear	Low	Low	Low	Low
Strandskov et al. (2017)	Low	Low	Unclear	Low	Low	Low	Low
Trompetter et al. (2015)	Low	Unclear	Unclear	Low	Low	Low	Unclear
van Aubel et al. (2020)	Low	Low	Low	Low	Low	Low	Low
Viskovich & Pakenham (2020)	Low	Low	Unclear	Low	Low	Unclear	Unclear
Weineland et al. (2012)	Unclear	Unclear	Unclear	Low	Low	Unclear	Unclear
Witlox et al. (2021)	Low	Low	High	Low	Low	Low	Unclear
